# Characterization of a Novel Functional Trimeric Catechol 1,2-Dioxygenase From a *Pseudomonas stutzeri* Isolated From the Gulf of Mexico

**DOI:** 10.3389/fmicb.2020.01100

**Published:** 2020-06-04

**Authors:** Julieta Rodríguez-Salazar, Arisbeth G. Almeida-Juarez, Katya Ornelas-Ocampo, Sofía Millán-López, Enrique Raga-Carbajal, José Luis Rodríguez-Mejía, Luis Felipe Muriel-Millán, E. Ernestina Godoy-Lozano, Nancy Rivera-Gómez, Enrique Rudiño-Piñera, Liliana Pardo-López

**Affiliations:** Instituto de Biotecnología, Universidad Nacional Autónoma de México, Cuernavaca, Mexico

**Keywords:** *Pseudomonas*, intradiol dioxygenase, aromatic compounds, catechol degradation, trimeric structure

## Abstract

Catechol 1,2 dioxygenases (C12DOs) have been studied for its ability to cleavage the benzene ring of catechol, the main intermediate in the degradation of aromatic compounds derived from aerobic degradation of hydrocarbons. Here we report the genome sequence of the marine bacterium *Pseudomonas stutzeri* GOM2, isolated from the southwestern Gulf of Mexico, and the biochemical characterization of its C12DO (PsC12DO). The *catA* gene, encoding PsC12DO of 312 amino acid residues, was cloned and expressed in *Escherichia coli.* Many C12DOs have been described as dimeric enzymes including those present in *Pseudomonas* species. The purified PsC12DO enzyme was found as an active trimer, with a molecular mass of 107 kDa. Increasing NaCl concentration in the enzyme reaction gradually reduced activity; in high salt concentrations (0.7 M NaCl) quaternary structural analysis determined that the enzyme changes to a dimeric arrangement and causes a 51% decrease in specific activity on catechol substrate. In comparison with other C12DOs, our enzyme showed a broad range of action for PsC12DO in solutions with pH values ranging from neutral to alkaline (70%). The enzyme is still active after incubation at 50°C for 30 min and in low temperatures to long term storage after 6 weeks at 4°C (61%). EDTA or Ca^2+^ inhibitors cause no drastic changes on residual activity; nevertheless, the activity of the enzyme was affected by metal ions Fe^3+^, Zn^2+^ and was completely inhibited by Hg^2+^. Under optimal conditions the *k*_*cat*_ and *K*_*m*_ values were 16.13 s^–1^ and 13.2 μM, respectively. To our knowledge, this is the first report describing the characterization of a marine C12DOs from *P. stutzeri* isolated from the Gulf of Mexico that is active in a trimeric state. We consider that our enzyme has important features to be used in environments in presence of EDTA, metals and salinity conditions.

## Introduction

Aromatic compounds are principal contaminants, and these show high toxicity for humans and animals (Costantini et al., 2009). For this reason, strategies have been sought to remove them from the environment (Yagi, 1985).

Several microorganisms are capable of degrading aromatic compounds such as catechols in a wide number of environments by using them as sources of carbon and energy. The genus *Pseudomonas* includes strains able to metabolize organic pollutants, crude oil components, and more complex aromatic compounds. The genomic size variation (3.7–7.1 Mb) in these species could explain their ability to use different organic compounds for growth (Singh et al., 2016).

In the aerobic biodegradation of aromatic compounds, catechols are of interest as central intermediates in the degradation pathway (Harwood and Parales, 1996; Ladino-Orjuela et al., 2016). These intermediates are subsequently degraded by catechol dioxygenases (CDOs), which cleave the aromatic ring by the introduction of two atoms of oxygen. The cleavage can occur in two positions: *ortho* or intradiol, with cleavage occurring between carbons 1 and 2 (C12DO) relative to the hydroxyl substituents, and *meta* or extradiol cleavage, between carbons 2 and 3 (C23DO). The final products of these two pathways enter the tricarboxylic acid (TCA) cycle (Harayama et al., 1992).

Members of the C12DO family (EC1.13.11.1) have been purified and characterized mainly in Gram-negative bacteria (Nakai et al., 1990; Shumkova et al., 2009; Sridevi et al., 2012). C12DOs present a non-heme ferric ion ligated by two histidines and two tyrosines in its catalytic site, showing molecular masses from 22 to 35 kDa and a high substrate specificity to catechol and some catechol derivatives (Wang et al., 2006; Krastanov et al., 2013; Al-Hakim et al., 2015). C12DOs are reported as dimeric enzymes, with special features as the ‘helical zipper’ formed by five N-terminal helices from each subunit with the presence of bound lipids at the dimeric interface (Vetting and Ohlendorf, 2000; Earhart et al., 2005).

The first C12DO purified was obtained from *Pseudomonas fluorescens* (Hayaishi et al., 1957) followed by C12DO of *Pseudomonas arvilla C-1* (*Pseudomonas putida*) (Nakai et al., 1979), this last have two non-identical subunits α and β of C12DO, that can form homo- and hetero- dimers (Nakai et al., 1979, 1990). In some strains of *Pseudomonas* genus, exist just one copy of C12DO as in the case of *Pseudomonas chlororaphis* strain UFB2 and *Pseudomonas aeruginosa* TKU002, where some characteristics as size (smaller size in C12DO from *P. aeruginosa* TKU002 22 kDa) and substrate specificity varies, but the dimeric arrangement is highly preserved (Wang et al., 2006; Setlhare et al., 2019).

C12DOs from *Acinetobacter* sp., *Burkholderia* sp., and *Pseudomonas* sp. were crystallized in different complexes with substrates other than catechol (ex. 1,2-ethanediol), which confirm that this enzyme can bind a variety of substrates in its catalytic site (Vetting and Ohlendorf, 2000; Earhart et al., 2005; Micalella et al., 2011, PDB entries: 2XSR 5UMH, 5TD3, and 5VXT). An alternative to the dimeric state in C12DOs was discovered in *Acinetobacter radioresistens*, in which Iso A forms an active trimer in solution under low ionic strength conditions (Briganti et al., 1997, 2000), representing the first example of a C12DO in which the oligomeric state is affected by the biochemical environment.

In this work, we report the genome of a marine *P. stutzeri* isolated from the southwestern Gulf of Mexico (swGoM) and describe the first C12DO (PsC12DO, EC 1.13.11.1) in the *Pseudomonas* genus active as a trimer, with a broad range of activity in the presence of different ions and pH values to be used in environments in presence of EDTA, metals and salinity conditions like industrial waste waters.

## Materials and Methods

### Isolation of the GOM2 Strain

In September of 2015, a seawater sample from the swGoM was collected in the Perdido Escarpment area (25°49′48.0′′N 95°30′00.0′′W) at a depth of 1,000 m using a CTD Rosette sampler (10 L Niskin bottles, previously sterilized with ethanol). One milliliter from the seawater sample was used to inoculate 30 mL of liquid (PY) medium composed of peptone and yeast extract (5 g/L, each) and 1% Bacab alfa crude oil as an additional carbon source. The culture was incubated in a cold room for 8 weeks. One hundred microliters from the culture was used for a serial dilution procedure with the aim of obtaining bacterial isolates, and a 10^–5^ dilution was spread onto LB agar plates and incubated for 3 days at 30°C. Several morphologically distinct colonies were observed. Only unique phenotypes were selected, and then each isolate was sprayed with a 5% catechol solution and incubated; within a few minutes (less than 10 min), orange/brown color formation was considered indicative of dioxygenase activity (Zukowski et al., 1983; Edlund and Jansson, 2006). One member of the *Pseudomonas* genus was selected as a potential candidate for this activity.

### Nucleic Acid Extraction and Sequencing

An isolated colony from the strain GOM2 was cultured in LB liquid medium at 30°C overnight, and the total DNA was extracted using the Quick-DNA^TM^ Miniprep Kit from Zymo Research (Irvine, CA, United States) following the kit recommendations. A sequencing library was prepared for the Illumina NextSeq 500 platform following the vendor’s protocol (San Diego, CA, United States).

### Genome Assembly and Annotation

A total of 7,106,750 paired reads (533,006,250 bases) were used for *de novo* genome assembly with ABySS v1.5.2 (Simpson et al., 2009) with a *k*-mer size of 41 after filtering for raw sequences with quality ≥Q20. To improve the high-quality draft genome, we used REAPR v1.0.18 (Hunt et al., 2013) for misassembly error correction, BESST v2.2.5 (Sahlin et al., 2014) for scaffolding, GapFiller v1-10 (Nadalin et al., 2012) for scaffold gap filling and iCORN 2 v0.95 (Otto et al., 2010) for scaffold correction. Functional annotation was performed with an adaptation of the Trinotate pipeline^1^ after gene prediction with GeneMarkS v4.32 (Borodovsky and Lomsadze, 2014). Genome completeness and contamination were performed with CheckM v1.0.12 (Parks et al., 2015).

### Genome Taxonomy and Phylogenetics

Taxonomic annotation was performed employing Kraken version 2.0.7-beta (Valenzuela-González et al., 2016) and MetaPhlAn2 v2.2.0 (Truong et al., 2015). The 16S rRNA gene was predicted with RNAmmer v1.2 (Lagesen et al., 2007) and annotated by BLAST with the NCBI Bacterial 16S Ribosomal RNA RefSeq database (BioProject PRJNA33175), RDP (Cole et al., 2014), SILVA ribosomal RNA gene database (Quast et al., 2012) and Greengenes database (DeSantis et al., 2006). Whole-genome comparison and determination of ANI/AAI values were performed using the Microbial Genome Atlas (MiGA) (Rodriguez-R et al., 2018) and NCBI prokaryotic databases. An ANI heatmap was generated with pyani (Pritchard et al., 2015) using all the genomes of *P. stutzeri* available as of Nov 2019 in GenBank, including *Pseudomonas* sp. R2 (Supplementary Table S1).

### Bacterial Strains, Plasmids, and Culture Conditions

Plasmids and strains used in this study are listed in Table 1. *Pseudomonas* strains were cultured at 30°C in mineral medium with the following composition in g/L: 0.8 K_2_HPO_4_, 0.2 KH_2_PO_4_, 0.3 NH_4_Cl, 0.19 Na_2_SO_4_, 0.07 CaCl_2_, 0.005 FeSO_4_.7H_2_O, 0.16 MgCl_2_, and 0.0002 Na_2_MoO_4_, as reported by Muriel-Millán et al. (2019), but supplemented with 2% sucrose. For *E. coli* cells, we used Luria–Bertani (LB) medium at 37°C (Miller, 1972), and the ampicillin concentration was 100 μg/mL.

**TABLE 1:** Bacterial strains and plasmids.

	Genotype/relevant characteristics	References
**Strains**		
*P. putida* KT2440	Wild-type strain mt-2 derivative cured of the TOL plasmid pWW0	Bagdasarian et al., 1981
*P. stutzeri* GOM2	Strain isolated from swGoM	This work
*E. coli* DH5ɑ	*supE44*Δ*lacU169 hsdR17 recA1 endA1 gyrA96 thi-1 relA1*	GIBCO-BRL
*E. coli* BL21(DE3)	*F - ompT hsdS B* (*r - B m - B*) *gal dcm*	Invitrogen
BL21(DE3)/pet22b+	*E. coli* BL21 carrying the empty expression vector pET22b+	This work
BLpETCDOPs	*E. coli* BL21 carrying the plasmid pETCDOPs for C12DO protein expression	This work
**Plasmids**		
pET22b+	Cloning and expression vector Amp^*r*^ His-tag	Novagen
pETCDOPs	pET22b + containing *catA* gene from *P. stutzeri* GOM2	This work

### Identification and Comparison of Catechol Dioxygenases From *Pseudomonas* Species

The sequences of a C23DO from *Pseudomonas alkylphenolica* (EC 1.13.11.2) and C12DO from *P. putida* (EC 1.13.11.1) (Zeyer et al., 1986) were used to search for dioxygenase genes in the genome of *P. stutzeri* GOM2 using BLASTP. Just one putative C12DO sequence was found with 73.72% percent identity and 99% coverage. The identity of the sequence was verified in the non-redundant (nr) database through BLASTP.

### Catechol Dioxygenase Phylogeny

A phylogeny was made with the sequence of amino acids identified as close homologs in the nr database for *Pseudomonas* sp. R2A2, and we also included additional C12DO sequences reported by Caglio et al. (2009), Guzik et al. (2013), and Nazmi et al. (2019) (Figure 2 and Supplementary Table S2). Evolutionary analyses were conducted in MEGA X (Kumar et al., 2018) with the maximum likelihood statistical method, 1,000 bootstrap replications and the Jones–Taylor–Thornton (JTT) model (Jones et al., 1992).

### Operon for Benzoate Catabolism and Genomic Context Identification

Additional degradation enzymes in metabolic pathways were searched, including aminobenzoate, naphthalene, fluorobenzoate, benzoate, and others, with a percent identity threshold of 50% using BLASTP and KEGG Mapper (Kanehisa and Sato, 2019). Eight enzymes involved in the benzoate degradation pathway were identified in the same C12DO genomic context (Supplementary Table S3 and Figure 3A).

### Induction of the *Ortho* Pathway in *P. stutzeri* GOM2

*Pseudomonas stutzeri* GOM2 was grown in mineral medium supplemented with 1 mM benzoic acid (BA) (Sigma, United States) or sucrose (BS) (J. T. Baker). The cultures were incubated overnight at 30°C and agitated at 200 rpm. Cells were harvested and centrifuged at 4,000 rpm for 15 min at 4°C, resuspended in phosphate buffer, pH 7.4, and sonicated. Samples containing 20 μg of total protein from the crude extracts were assayed for *cis*,*cis*-muconic acid (ccMA) production with 50 μM catechol as substrate, monitoring changes in product formation at 260 nm. *P. putida* KT2440 was used as a positive control. Protein concentration was determined by the Bradford method (Bradford, 1976) using bovine serum albumin (Sigma, United States) as a standard.

### Cloning of *catA* From *P. stutzeri* GOM2

The *catA* gene was amplified by PCR utilizing genomic DNA from *P. stutzeri* GOM2 as a template. The primers used were designed as FDO*EcoRI*stutz (5′- TTG TAG AAT TCA TGA CTG TGA AAA TCT CCC ACA C -3′) and RDO*XhoI*stutz (5′- AAC ATC TCG AGT CAG GCT TGC TGC AGT GC -3′) to amplify a predicted DNA fragment of 936 bp. Underlined sequences indicate *Eco*RI and *Xho*I restriction sites, respectively. The PCR product and pET-22b+ (Thermo Scientific, United States) were digested with *Eco*RI and *Xho*I, then ligated to generate the recombinant plasmid pET-22b+ *catA*, designated pETCDOPs. *E. coli* BL21(DE3) cells (Invitrogen, United States) were transformed by electroporation using this plasmid. Recombinant cells were confirmed by PCR, enzymatic digestion and sequencing.

### Overexpression and Purification of Recombinant PsC12DO

BL21 cells carrying the recombinant pETCDOPs plasmid were inoculated in LB medium containing 100 μg/mL ampicillin and grown at 37°C. An aliquot of the culture was inoculated into 100 mL of fresh medium. IPTG at 1 mM was added to the culture when the OD_600 *nm*_ reached 0.6 and further incubated for 2 h; after this time, cell culture was harvested by centrifugation at 4,000 rpm for 20 min at 4°C. Cells were washed with 50 mM phosphate buffer, pH 8.0, and disrupted by sonication. Expression of the *catA* gene in *E. coli* cells resulted in the production of the *P. stutzeri* GOM2, C12DO, named PsC12DO, carrying a 6 × His tag fused at the C-terminus. Purification of PsC12DO was carried out with the supernatant by Ni^2+^ affinity chromatography with Ni-NTA resin (Qiagen, Germany) under native conditions. Enzyme eluents were concentrated and ultrafiltered using Amicon Ultra-0.5 (Merk, Germany) with 50 mM glycine-NaOH buffer, pH 8.5, at 4°C. Enzyme purity was verified by SDS-PAGE. An acrylamide electrophoresis gel under non-denaturing conditions was performed (Supplementary Figure S2). Without SDS in running buffer and gel preparation, and reducing agents absent in samples to avoid denaturing conditions. Both samples of PsC12DO obtained in SEC (dimer and trimer) were mixed and loaded in gel. As standard, we used KatG from *Mycobacterium tuberculosis −80 kDa monomer* and *160 kDa dimer* (donated by Xavier Soberon’s Group IBt, UNAM).

### Size Exclusion Chromatography (SEC)

Size exclusion chromatography was performed using a HiLoad^TM^ 26/60 Superdex^TM^ 200 column (GE Healthcare Life Sciences) on an Äkta prime - FPLC System. For purification, the column was pre-equilibrated with 50 mM glycine-NaOH buffer, pH 8.5, and for seawater salinity conditions with 50 mM glycine-NaOH buffer, pH 8.5, and 700 mM NaCl. The sample was loaded onto the column (500 μL of protein at a concentration of 20 mg/mL). The purification was performed at a flow rate of 1.0 mL/min and the absorbance was measured at 280 nm.

The column was previously calibrated with four proteins as standards, bovine serum albumin - 66 kDa monomer/132 kDa dimer (SIGMA-ALDRICH), MBP-HilD −140 kDa (donated by Victor Bustamante’s Group, IBt, UNAM), Laccase from *Thermus thermophilus* −50 kDa (donated by Enrique Rudiño’s Group, IBt, UNAM), GFP −54 kDa (donated by Paul Gaytan’s Group, IBt, UNAM), and the mass of PsC12DO was determined by comparison the calibration curve.

### Standard Enzyme Assay

The activity of catechol 1,2-dioxygenase was measured spectrophotometrically using an Epoch 2 microplate reader (BioTek Instruments, United States) in 96-well UV-transparent microplates (Corning Inc., United States). The reaction mixture (200 μL) contained 50 mM glycine-NaOH buffer, pH 8.5, and 100 μM catechol. After microplate preincubation at 40°C, the reaction was started by adding the substrate. The ccMA formation rate was determined by monitoring the reaction at 260 nm for 3 min, employing a calibration curve with ccMA standard solutions (5–100 μM). One unit of dioxygenase activity (U) was defined as the amount of enzyme that catalyzed the formation of 1 μmol of product per min. All experiments were carried out in triplicate.

### Effects of pH and Temperature

The optimum pH for the PsC12DO enzyme was determined using the following buffers: 50 mM sodium acetate (pH 3.6–5.0), 50 mM Sorensen (pH 6.0–8.5), and 50 mM glycine-NaOH (pH 8.5–12). The activity was determined at 30°C. The optimum temperature was assayed at temperatures ranging from 15 to 60°C. The reactions were performed in 1-cm quartz cuvettes and followed using a Beckman DU-640 spectrophotometer (Beckman Coulter, United States). To evaluate enzyme thermostability, 1 mL of enzyme sample (40 μg/mL) was incubated in 50 mM glycine buffer, pH 8.5, at 15, 20, 30, 35, 40, 45, 50, and 60°C using an Eppendorf ThermoMixer (Germany). After specific time intervals, 50-μL aliquots were withdrawn, chilled on ice, and used to determine residual enzyme activity.

### Effects of Metal Ions and Salinity

The impacts of various metal ions [Fe_2_(SO_4_)_3_, CaCl_2_, ZnSO_4_, CoCl_2_, KCl, CuSO_4_, FeSO_4_, MgCl_2_, MnSO_4_, NiCl_2_, HgCl_2_] and EDTA as a chelating agent on PsC12DO activity were evaluated by incubating the enzyme (1.3 μg/mL) with these compounds at 1.3 mM for 15 min at 40°C and then assaying the residual activity by adding catechol to initiate the reaction.

To evaluate the effect of salinity on PsC12DO activity, enzymatic reactions were carried out in the presence of 50 mM glycine-NaOH buffer supplemented with NaCl concentrations ranging from 0.025 to 1.5 M as described in the standard enzyme assay.

### Determination of Kinetic Constants and Substrate Specificity

The catalytic parameters (*K*_*m*_, *k*_*cat*_, and *k*_*cat*_/*K*_*m*_) were determined by calculating the initial velocity of reactions varying catechol concentrations (0–150 μM) in 50 mM buffer glycine-NaOH, pH 8.5, at 40°C. The data were fitted by non-linear regression to the typical Michaelis–Menten equation employing the software GraphPad Prism version 6.01 (GraphPad Software Inc.). The *K*_*cat*_ value was calculated based on the molecular mass of the enzyme subunit (37.5 kDa).

To examine substrate specificity, the catechol derivatives 3-methylcatechol (3-MC), 4-methylcatechol (4-MC), and 4-chlorocatechol (4-CC) were assayed as substrates for PsC12DO. The reactions were performed with 100 μM substrate under standard enzyme assay conditions. The specific activities (μmol product/min/mg of protein) were calculated by employing the product molar extinction coefficients reported elsewhere (Dorn and Knackmuss, 1978).

### Hydrodynamic Diameter Determination by Dynamic Light Scattering (DLS)

The PsC12DO hydrodynamic diameter was determined by DLS analysis with the Zetasizer Nano Z system (Malvern Panalytical). The protein sample (1 mg/mL in a volume of 1 mL) in glycine-NaOH buffer, pH 8.5, was equilibrated at 40°C for 1 min. The size values reported are an average of 3 runs per measurement with a scattering angle of 173°. All measurements were carried out in triplicate. Samples were characterized in the absence and presence of salt (0 and 700 mM NaCl).

### Structural Sequence Analysis

Sequence alignment was generated by Clustal Omega (Madeira et al., 2019) using full-length C12DO sequences homologous to PsC12DO; these sequences were obtained from a pre-analysis performed by FASTA-EBI (Madeira et al., 2019). Final alignment was made by ESPript 3.0 (Robert and Gouet, 2014). A model of PSC12DO was elaborated using the CPHmodels 3.2 Server based on the C12DO *P. arvilla* structure (PDBid: 2AZQ) (Nielsen et al., 2010), and the structural figure was generated using PyMOL.

### Statistical Analysis

Statistical analysis was conducted using GraphPad Prism version 6.01 (GraphPad Software Inc.) by ANOVA test (*p* < 0.01).

## Results

### Isolation and Identification of the GOM2 Strain

To isolate aromatic compound-degrading bacteria from the Gulf of Mexico, a water-column sample was inoculated into liquid PY medium with crude oil as an additional carbon source (see section “Materials and Methods”). Three different isolates were identified according to their colony morphology in LB plates, of which two exhibited a reddish-brown color after catechol application, suggesting catechol transformation (Edlund and Jansson, 2006). The isolate that showed the darkest brown color was selected and named the GOM2 strain.

### Genome Assembly, Taxonomic Annotation, and Phylogenetic Analysis

**FIGURE 1:**
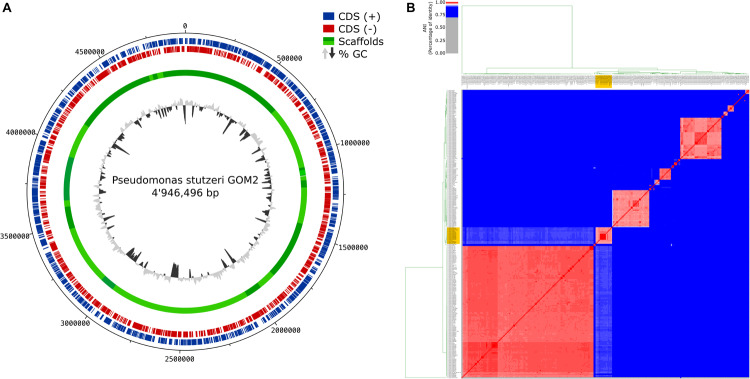
Genome assembly and ANI comparison. **(A)** Circular diagram of *P. stutzeri* GOM2 showing (from inner to outer)% G + C, GC skew and scaffolds. **(B)** Average nucleotide identity heatmap with all the available *P. stutzeri* genomes from GenBank. The yellow box indicates the group to which *P. stutzeri* GOM2 is related.

The GOM2 genome was assembled with a total of 7,106,750 sequences with an average quality of 33.59. The improved assembly included 24 scaffolds with an N50 of 419,118 bp contained in four scaffolds and an N90 of 114,516 bp contained in 12 scaffolds. A total of 96.75% of the raw reads were mapped to the assembly, and 93.75% were properly paired and mapped. The final genome size was 4.94 Mb with a G + C content of 63.54% and 108× coverage (Figure 1A). Genome completeness was 100%, and contamination was 9%. Raw sequences have been made available in the SRA database under accession number PRJNA592574, and the genome has been deposited into DDBJ/ENA/GenBank under accession number WOUM00000000. The version described in this paper is version WOUM01000000.

The taxonomic annotation was confirmed with different methods. Eight copies of the 16S rRNA gene were predicted to have 99.8% identity with *P. stutzeri* annotated with different 16S rRNA databases (Supplementary Table S4). In addition, 96.64% of the sequences coincided with a *k*-mer spectrum of *Pseudomonas* at the genus level. Single-copy markers classified it as *P. stutzeri* unclassified (99.37% sequences), and ANI/AAI indicated *Pseudomonas* sp. R2A2 (97.61% ANI and 98.4% AAI) (Figure 1B).

To compare the GOM2 strain at the complete-genome level, we calculated the average nucleotide identity of the 277 *P. stutzeri* genomes available in GenBank at the date of manuscript submission and included the *Pseudomonas* sp. R2A2 strain, which MiGA identified as the most correlated strain via the NCBI Prok database. The GOM2 strain was closest to the following strains: *Pseudomonas* sp. R2A2, *P. stutzeri* UBA7601, *P. stutzeri* UBA5710, *P. stutzeri* ATCC 17588, *P. stutzeri* HI00D01, *P. stutzeri* UBA1598, *P. stutzeri* UBA1470, *P. stutzeri* UBA1468, *P. stutzeri* UBA6501, *P. stutzeri* UBA3984, *P. stutzeri* UBA6599, *P. stutzeri* UBA2499, *P. stutzeri* DNSP21, *P. stutzeri* UBA1495, and *P. stutzeri* PS_167. The ANI percentage range was between 96.60 and 97.65% (Figure 1B).

### Identification and Comparison of Catechol Dioxygenases From *Pseudomonas* Species

We searched two dioxygenase genes previously reported in *Pseudomonas* genomes, but we did not find EC 1.13.11.2 in the GOM2 genome; we found only EC 1.13.11.1 with 73.72% percent identity and 99% coverage compared to a sequence from *P. putida* (A5W235). In the nr database, we found that this sequence was a C12DO and had high percent identity to sequences from other *Pseudomonas* strains and another *P. stutzeri* (Supplementary Table S2). The best hit was the TPA sequence: C12DO of *Pseudomonas* sp. (HAJ87005), to which it had 99% identity and 100% coverage. We searched nr excluding the *Pseudomonas* taxon and found that species such as *Acinetobacter*, *Marinobacterium*, *Marinobacter*, and *Streptococcus* had identity values of over 57% to the C12DO of *P. stutzeri* (Supplementary Table S2). This analysis corroborates that the sequence we identified using reference sequences is a C12DO.

### Catechol Dioxygenase Phylogeny

**FIGURE 2:**
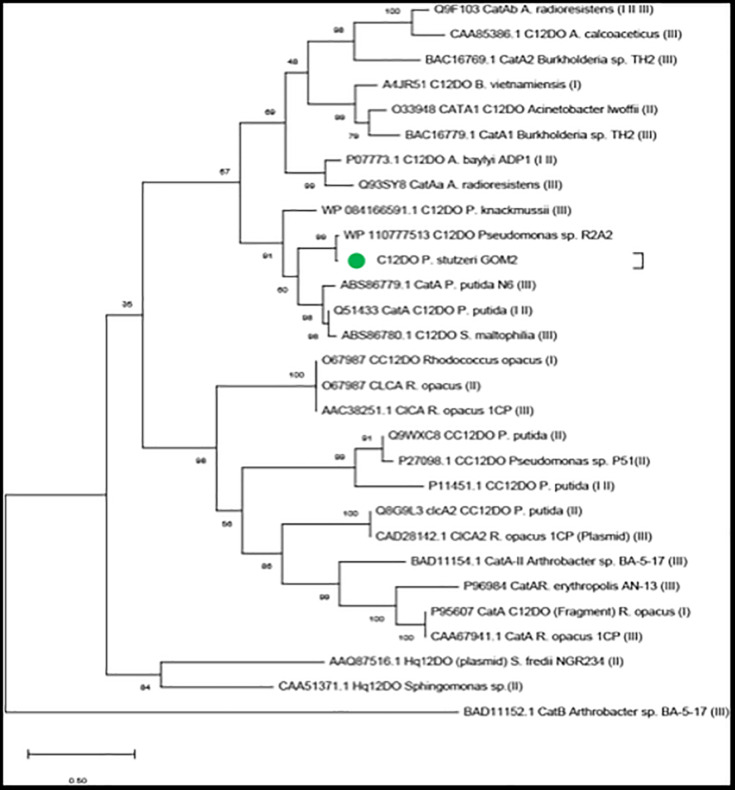
Evolutionary analyses. The green circle denotes the sequence of *P. stutzeri* GOM2. In the parentheses the reference is indicated where the protein sequence was obtained: (I) Nazmi et al. (2019), (II) Caglio et al. (2009), and (III) Guzik et al. (2013).

For evolutionary analysis of the C12DOs, we used the sequences identified in the nr database and the sequences listed by Caglio et al. (2009), Guzik et al. (2013), and Nazmi et al. (2019), which includes sequences of C12DOs, chlorocatechol 1,2-dioxygenases (CC12DOs) and hydroxyquinone 1,2-dioxygenase from different organisms (HQ12DOs) (Figure 2 and Supplementary Table S2). In the tree, C12DO from *P. stutzeri* GOM2 is grouped with *Pseudomonas* and *Stenotrophomonas maltophilia* C12DO sequences. This group contains C12DOs that bind substrates such as catechol but also 3-chlorocatechol (3-CC), 4-chlorocatechol (4-CC), 3-methylcatechol (3-MC) and 4-methylcatechol (4-MC), as in the case of *P. knackmussii* (before *Pseudomonas* sp. B13) (Guzik et al., 2013). These enzymes are type I catechol dioxygenases. The cladogram shows another group with sequences from the genera *Rhodococcus* and *Arthrobacter* that contains type II CC12DOs (Eulberg et al., 1997).

### Benzoate Catabolism Operon

**FIGURE 3:**
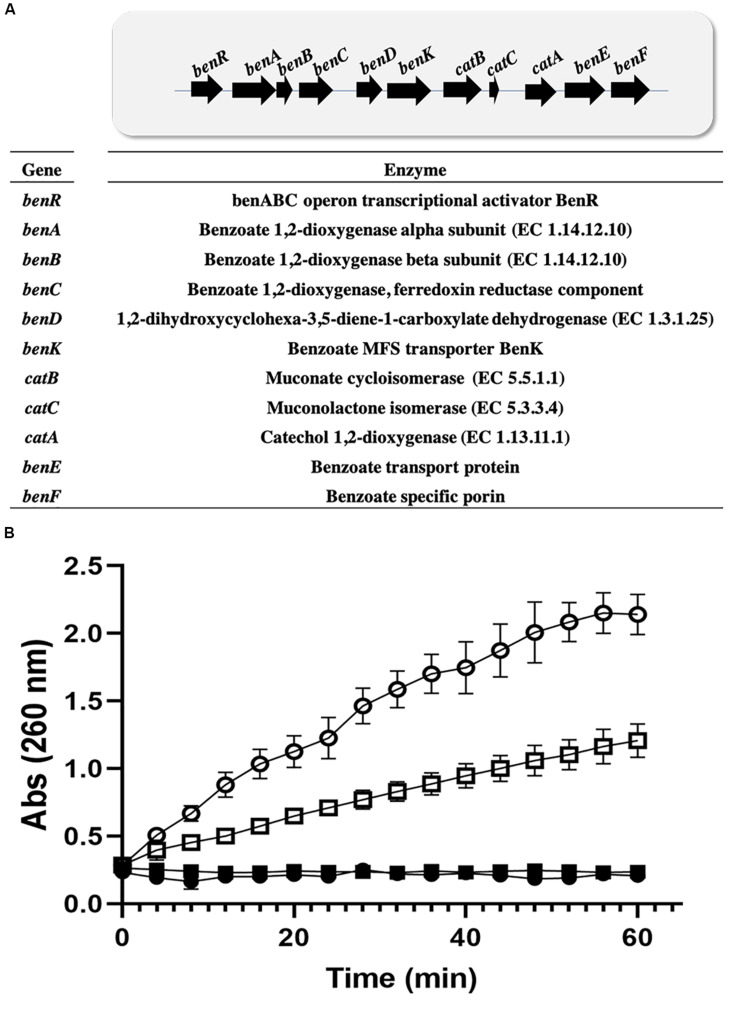
Induction of ortho pathway in *Pseudomonas.* **(A)** Benzoate catabolism operon in *P. stutzeri* GOM2. **(B)** *Cis*,*cis*-muconate production by *Pseudomonas* strain crude extracts. *P. stutzeri* GOM2 grown with BS (closed circles) or BA (open circles) and *P. putida* KT2440 grown with BS (closed squares) or BA (open squares) are shown. The assay was carried out in 96-well microplates, using 10 μg/mL total crude protein, with a 50 mM catechol solution in PBS buffer, pH 7.4, at 30°C. Values are the averages of three measurements, and bars indicate the SD values.

We used a collection of 300 enzyme sequences involved in the degradation of xenobiotics to search the genome of *P. stutzeri* for enzymes other than EC 1.13.11.1. Eight enzymes were identified from the benzoate pathway (Supplementary Table S5). The enzymes identified are in a continuous pathway and are also distributed in the same genomic context. The enzymes were identified in two scaffolds, 14 and 15 (Supplementary Table S5), on scaffold 15, where C12DO is located; 5 enzymes were found in the same context, corresponding to two EC 1.14.12.10 sequences, EC 1.3.1.25, EC 5.5.1.1, EC 5.3.3.4, and EC 1.13.11.1. In these genetic regions, regulatory proteins were identified: the *benABC* operon transcriptional activator IP BenR and a benzoate transporter, the MFS BenK transporter (Figure 3A). Enzymes involved in the degradation of aromatic compounds other than benzoate were not identified in GOM2 genome.

### Induction of C12DO in *Pseudomonas*, Cloning, and Purification

**FIGURE 4:**
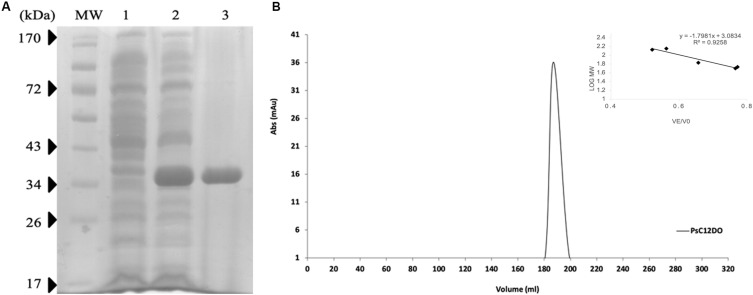
Size exclusion chromatography of PsC12DO. **(A)** SDS–PAGE analysis of purified PsC12DO. MW, molecular weight ladder. Lane 1, crude extract. Lane 2, crude extract after the addition of IPTG. Lane 3, purified PsC12DO. **(B)** Chromatogram showing the elution profile of PsC12DO at 180 mL (108 kDa). On the right, a five-point calibration graph.

Benzoic acid is converted to catechol and further degraded to ccMA by the action of dioxygenases. *P. stutzeri* GOM2 grew efficiently on 1–3 mM BA, and for this reason we investigated whether the *ortho* degradation pathway was induced when BA was added to culture media following the identification of the operon for benzoate catabolism. PsC12DO is an inducible enzyme, since no ccMA production was detectable in the crude extract when the GOM2 strain was grown in a medium containing a different source of carbon, such as sucrose (Figure 3B). The same was observed when *P. putida* was grown with BA, and no induction was observed when sucrose was the carbon source since ccMA was not detected (Figure 3B). The amplification of the *catA* gene resulted in a fragment of 936 bp, encoding a protein of 312 amino acid residues with a calculated molecular mass of 34.7 kDa, here named PsC12DO. The enzyme was successfully expressed in *E. coli* and purified to homogeneity (Figure 4A), with a fold purification of 8.4 and recovery of 42.5% (Table 2). As complement of the data obtained in quaternary structure, we performed an acrylamide electrophoresis gel under non-denaturing conditions where both states, dimer and trimer are observed (Supplementary Figure S2).

**TABLE 2:** Purification of PsC12DO.

Purification steps	Volume (mL)	Volumetric activity (U/mL)	Total activity (U)	Total protein (mg/mL)	Specific activity (U/mg)	Yield (%)	Purification fold
Crude extract	20	0.9	18.3	0.5	1.8	100	1
Purified enzyme	0.05	155.4	7.8	10.4	15	42.5	8.5

### Molecular Mass Estimation and Hydrodynamic Diameter of PsC12DO

PsC12DO molecular mass was estimated by SDS-PAGE under denaturing conditions resulting in a value of 35 kDa (Figure 4A). Additionally, PsC12DO was evaluated by size exclusion chromatography, and the protein eluted at 180 mL corresponding to a molecular mass of ∼107 kDa (Figure 4B); considering that the monomer size is 35 kDa, these data suggest that PsC12DO is a trimer. This size value was confirmed by DLS analysis, obtaining a hydrodynamic diameter of 10 nm (Supplementary Figure S1A).

### Effects of pH and Temperature on the Activity of PsC12DO

**FIGURE 5:**
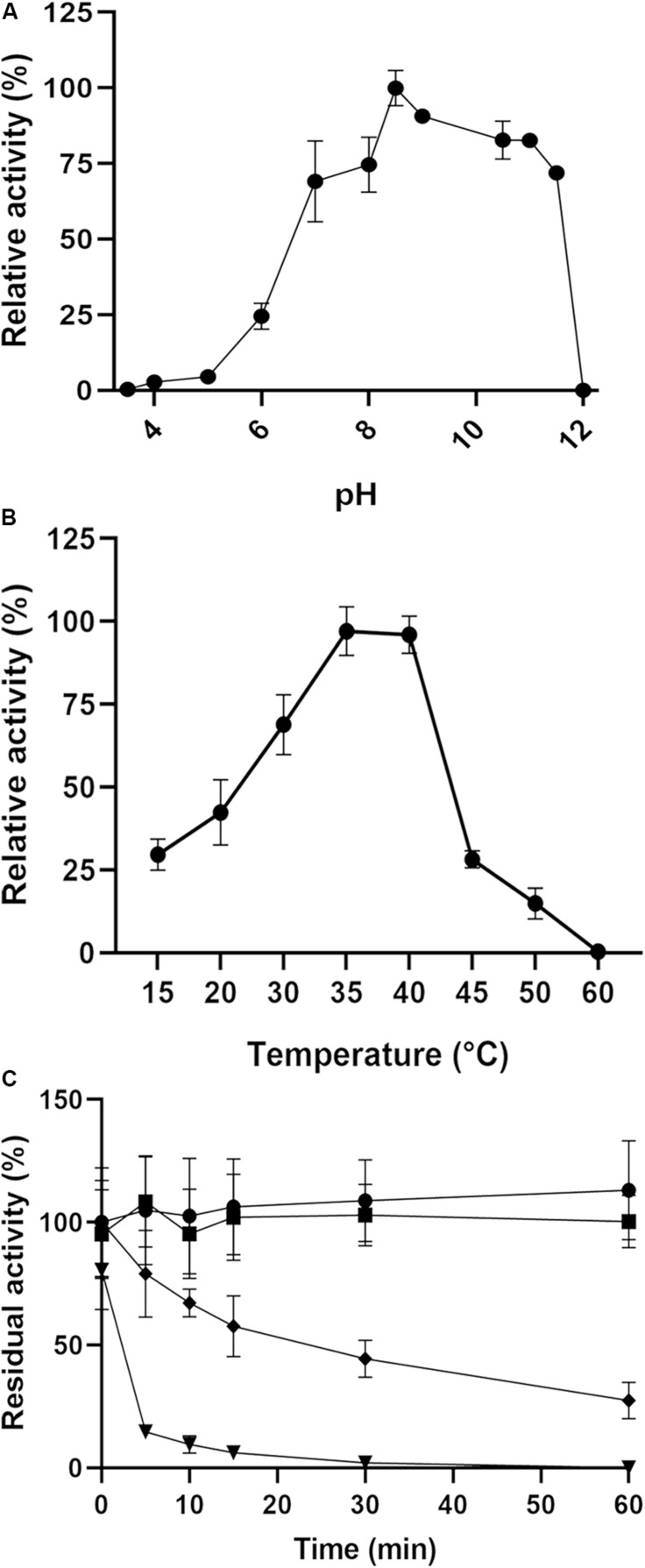
Effects of pH and temperature on the enzyme activity of PsC12DO. **(A)** Activity assays were performed in 50 mM sodium acetate (pH 3.6–5.0), 50 mM Sorensen (pH 6.0–8.5), and 50 mM glycine-NaOH (pH 8.5–12) at 40°C. **(B)** Activity assays were performed in a range of 15–60°C to determine the optimal temperature condition. **(C)** Thermal stability assays were performed in a range of 40–60°C. Temperatures of 40°C (circles), 45°C (squares), 50°C (diamonds) 60°C (triangles) in 50 mM glycine-NaOH buffer pH 8.5. Values are the average of three measurements, and bars indicate the SD values.

The optimal pH for enzyme activity was found to be approximately 8.5, and the enzyme maintained more than 70% activity over a broad pH range (7–11.5). At pH 6, the enzyme performance was severely affected, showing just 24% activity, and it was almost inactive at pH values below 5 and above 12 (Figure 5A). The activity was also examined at several temperatures ranging from 15 to 60°C, and an optimum temperature range of 35–40°C was determined. The enzyme retained 30% activity at 15 and 45°C but lost its activity at 60°C (Figure 5B). With regard to thermal stability, the activity was not affected at 40 or 45°C after 1 h of incubation; however, the enzyme lost 50% of its activity after 30 min of incubation at 50°C but retained 20% activity after 1 h (Figure 5C). At 60°C, 10 min of incubation was enough to abolish 90% of the activity, and total inactivation was reached after 30 min.

### Effects of Inhibitors, Substrate Specificity and Storage Stability

**FIGURE 6:**
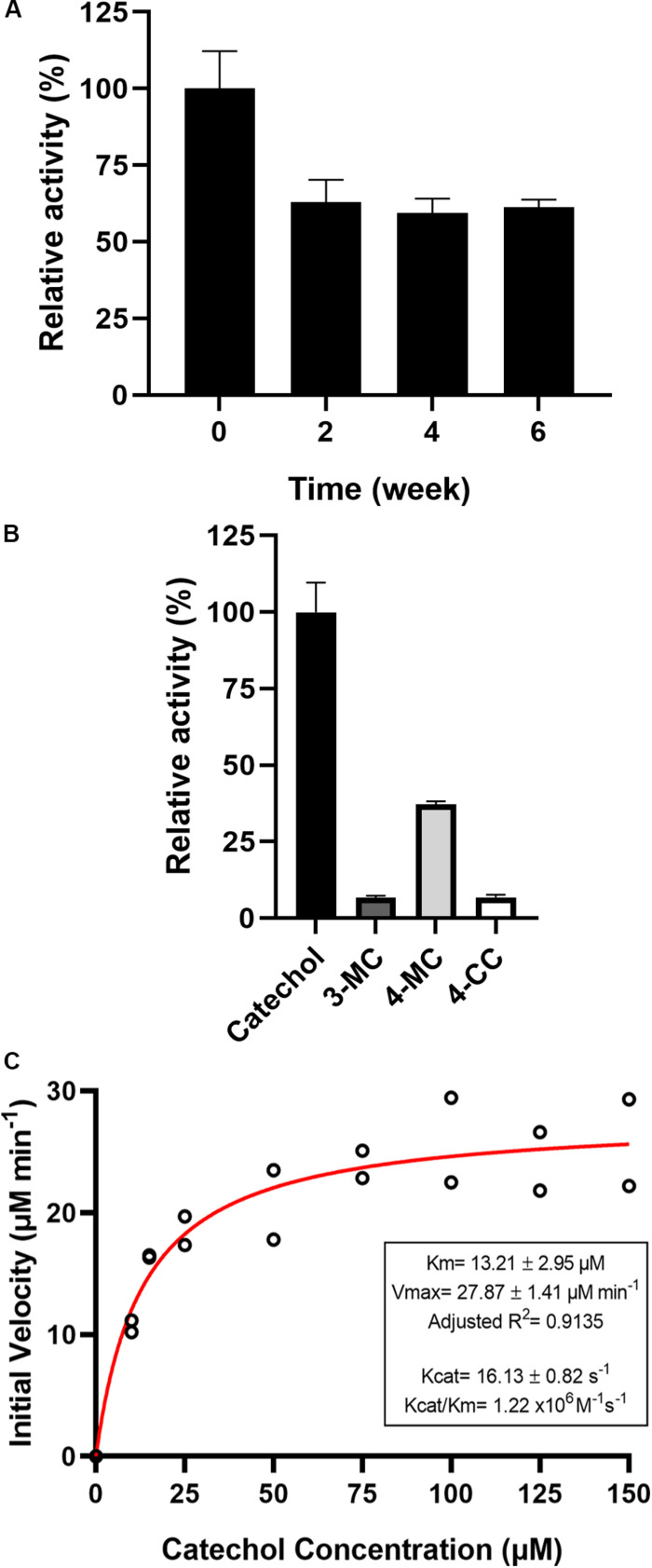
**(A)** Storage stability of PsC12DO at 4°C. **(B)** Substrate specificity of PsC12DO. Catechol (black), 3-MC (gray), 4-MC (light gray), and 4-CC (white) are shown. **(C)** Effect of catechol concentration on enzyme activity and kinetic parameters. Values are the averages of three measurements, and bars indicate the SD values.

The effects of metal ions and chelators were investigated. More than 70% of PsC12DO activity was retained after incubation for 30 min with 1.3 mM of Co^2+^, K^+^, EDTA, Fe^2+^, and Ni^2+^, whereas PsC12DO activity was reduced by approximately 50% by Ca^2+^, Cu^2+^, Mg^2+^ and Mn^2+^. Furthermore, the enzymatic reaction was severely affected by Fe^3+^ and Zn^2+^, showing inhibition of up to 90%, and was completely inhibited by Hg^2+^ (Table 3). The substrate specificity of PsC12DO was evaluated by employing three different substituted catechol derivatives: 3-MC, 4-MC, and 4-CC. The results showed 37% relative activity for 4-MC and minor relative activities of 6.7 and 6.8% for 3-MC and 4-CC, respectively (Figure 6B). Hence, PsC12DO has low specificity for substituted substrate derivatives. We also tested the stability of the purified enzyme after storage at 4°C; the enzyme retained 61% of its original activity after 6 weeks of storage (Figure 6A).

**TABLE 3:** Effect of metal ions and chelators on the enzyme activity of PsC12DO.

Compound	Relative activity (%)
None (control)	99.93 ± 4.43
Fe^3+^	4.21 ± 3.93^b^
Ca^2+^	61.44 ± 4.95
Zn^2+^	7.75 ± 0.38^b^
Co^2+^	76.38 ± 8.1
K^+^	72.42 ± 17.33
EDTA	71.22 ± 19.12
Cu^2+^	57.64 ± 11.43
Fe^2+^	80.05 ± 18.5
Mg^2+^	59.79 ± 7.52
Mn^2+^	48.68 ± 4.45^a^
Ni^2+^	72.26 ± 6.03
Hg^2+^	0.50 ± 0.05^b^

*Data are the means of three independent measurements ± SD. Different letters indicate statistical significance by multiple comparison (^*a*^p > 0.01, ^*b*^p > 0.001).*

### Enzyme Kinetics

To calculate the catalytic parameters *K*_*m*_, *k*_*cat*_, and *k*_*cat*_/*K*_*m*_ for catechol cleavage, the initial rates of the reactions catalyzed by PsC12DO were measured at different substrate concentrations (Figure 6C). The *K*_*m*_ and *k*_*cat*_ values obtained by non-linear regression fitting were 13.2 ± 2.95 μM and 16.13 ± 0.81 s^–1^, respectively, as well as a catalytic efficiency (*k*_*cat*_/*K*_*m*_) of 1.22 × 10^6^ M^–1^s^–1^. The saturation curve did not indicate any substrate inhibition of the enzyme in the substrate range studied.

### Effects of Salinity on PsC12DO Activity and Quaternary Structure

**FIGURE 7:**
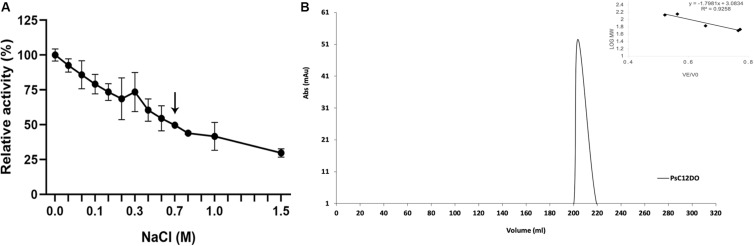
Effects of NaCl on the enzyme activity of PsC12DO. **(A)** Activity assays were performed in glycine-NaOH buffer, pH 8.5, supplemented with NaCl at 40°C. Values are the average of three measurements, and bars indicate the SD values. **(B)** Chromatogram showing the elution profile of PsC12DO at 203 mL (89 kDa) in 50 mM glycine-NaOH, pH 8.5, supplemented with 700 mM NaCl. On the right, a five-point calibration graph.

The relative activity of PsC12DO was tested when subjected to different salt concentrations. Salinity gradually reduced the enzyme activity, but it retained 54% of its original activity at 0.5 M and 30% at 1.5 M (Figure 7A). PsC12DO quaternary structure was evaluated under salinity conditions, with 50 mM glycine-NaOH buffer, pH 8.5, supplemented with 700 mM NaCl; under these conditions (similar to seawater salt concentration), PsC12DO presented an elution volume of 203 mL, which corresponds to a molecular mass of ∼89 kDa (Figure 7B) and a hydrodynamic diameter of 8 nm, showing the dimeric state of the enzyme (Supplementary Figure S1B).

### PsC12DO Sequence Analysis and Structural Model

**FIGURE 8:**
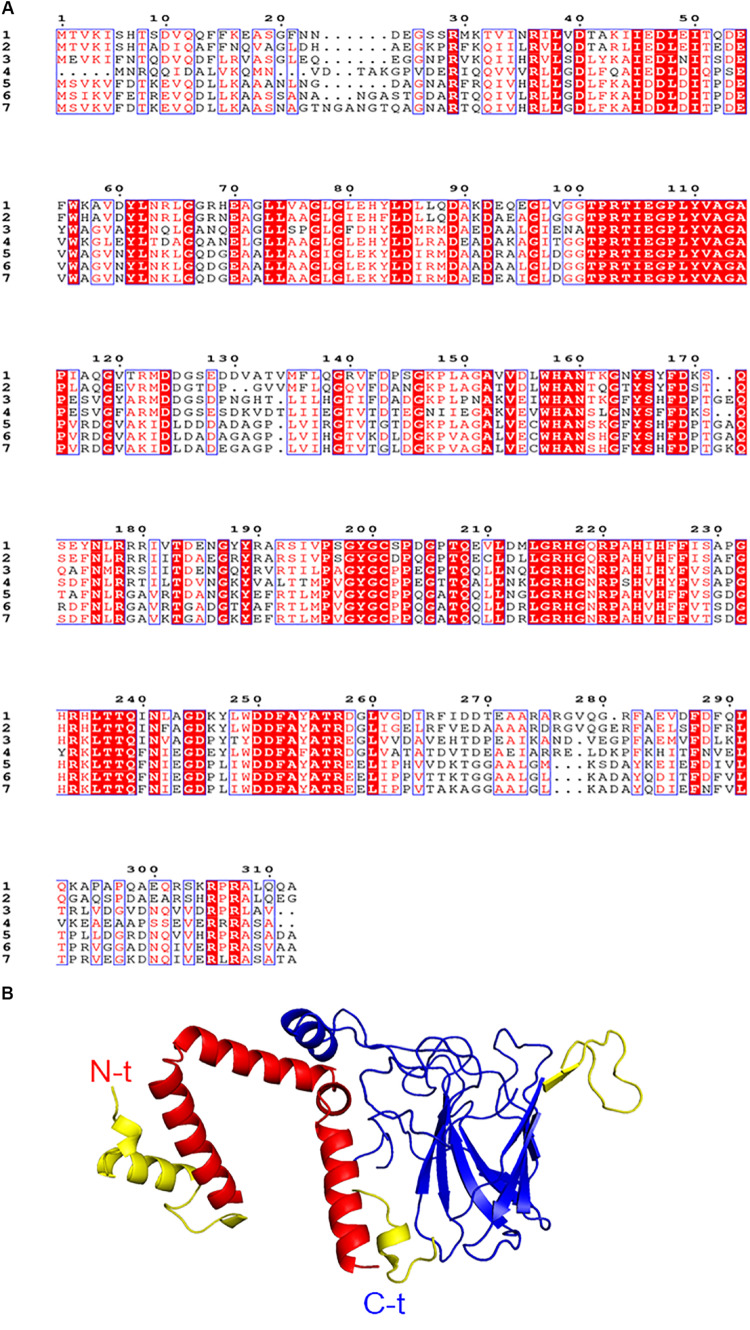
**(A)** Amino acid sequence alignment of six catechol 1,2-dioxygenase homologs to PSC12DO. Residues in red are conserved in all sequences. Residues in a blue frame have similarity. (1) Catechol 1,2-dioxygenase *Pseudomonas stutzeri* (PsC12DO), (2) Catechol 1,2-dioxygenase *Pseudomonas Arvilla* (*PDBid:2AZQ*), (3) Catechol 1,2-dioxygenase *Acinetobacter calcoaceticus* (*PDBid:1DLM*), (4) Catechol 1,2-dioxygenase *Acinetobacter radioresistens* (*PDBid:2XSR*), (5) Catechol 1,2-dioxygenase *Burkholderia multivorans* (*PDBid:5UMH*), (6) Catechol 1,2-dioxygenase *Burkholderia vietnamiensis* (*PDBid:5TD3*), (7) Catechol 1,2-dioxygenase *Burkholderia ambifaria* (*PDBid:5VXT*). The alignment was generated by using Clustal Omega. **(B)** A model of monomeric PsC12DO generated by CHPModel 3.2 using the structure PDBid:2AZQ as reference.

The alignment shows all sequences of C12DO homologs to PsC12DO with three-dimensional structure determined (Figure 8A). All residues from the catalytic site—*Y165*, *Y199*, *H223*, and *H225*—are conserved. The highest sequence identity percent is 73% with the *P. arvilla C-1* sequence (*PDBid:2AZQ*), followed by C12DOs from *Acinetobacter calcoaceticus*, 53% (*PDBid:1DLM*); *A. radioresistens*, 49% (*PDBid:2XSR*); *Burkholderia multivorans*, 48% (*PDBid:5UMH*); *Burkholderia vietnamiensis*, 48% (*PDBid:5TD3*); and *Burkholderia ambifaria*, 43% (*PDBid:5VXT*). The most variable regions are located in residues 1–29 from the N-terminus and the regions 265–282 and 291–312 in the C-terminus; these regions can be observed in the PsC12DO monomeric model (Figure 8B).

## Discussion

### Phylogenetics and Bioinformatics of *P. stutzeri* GOM2

Through taxonomic characterization, we confirmed that the strain GOM2 belongs to the species *P. stutzeri*, as demonstrated by several methods, including *k*-mer, single-copy markers and 16S rRNA gene prediction. Considering the results obtained by ANI via MiGA and comparing all available genomes of *P. stutzeri* in GenBank, the two strains with the highest ANI proportions were *Pseudomonas* sp. R2A2, with an ANI of 97.65%, and *P. stutzeri* UBA7601, with an ANI of 97.37%. *Pseudomonas* sp. R2A2 (GenBank accession: CP029772.1) was isolated from groundwater in Taiwan, and this strain has a role in arsenic transformation reactions. *P. stutzeri* UBA7601 (GenBank accession: GCA_002477745.1) is a genome isolated from a metagenomic sample of wood in New York City. The high resemblance and genome percentages shared with these strains highlight the high plasticity of the *Pseudomonas* genus, which allows it to adapt to different environments.

PsC12DO is a type I dioxygenase enzyme that shows high identity with other enzymes in the same group, such as the dioxygenase of *P. putida*. This enzyme is found in genomic synteny with other enzymes implicated in the degradation of benzoate. Bioinformatically, we can speculate that this bacterium probably degrades benzoate in the pathway from benzoate to succinyl-CoA, which would integrate into the TCA cycle, because its genome contains the necessary genes that encode the enzymes to carry out this degradation.

### Effects of pH and Temperature on PsC12DO Activity

*Pseudomonas* dioxygenases play essential roles in the degradation of aromatic compounds. Many aromatic compounds are degraded to less toxic compounds by the action of these enzymes using oxygen by the *ortho* or *meta* pathway (Harayama and Rekik, 1989). In this paper, we report the biochemical characterization of a trimeric intradiol aromatic ring-cleavage dioxygenase from the *P. stutzeri* GOM2 strain (PsC12DO) isolated from the swGoM.

Factors such as pH, temperature and salinity are important determinants for optimal enzyme conditions, and these elements are taken into account when considering the efficiency and quantity of pollutant biodegradation by bacterial enzymes. A variation of pH affects catalytic reactions and enzyme activities (Bonomo et al., 2001; Krachler et al., 2009). Our results showed a broad range of action for PsC12DO in solutions with pH values ranging from neutral to alkaline (7.5–11.5, Figure 5A). These results are similar to those reported by Giedraityte and Kalėdienė (2009) for a C12DO from *S. maltophilia*, which was active over a broad range from pH 7 to 10.5 but not at pH 5. However, several reports indicate high activity of C12DOs at pH values of approximately 7 (Briganti et al., 2000; Guzik et al., 2011; Long et al., 2016) and low or absent activity at pH values below 5 (Silva et al., 2013; Long et al., 2016).

Temperature highly influences the composition of aromatic hydrocarbons (Atlas, 1981). Biodegradation of these compounds occurs over a wide range of temperatures; however, the biodegradation rate decreases at low temperatures as a result of reduced enzymatic activities. Most mesophilic enzymes are not highly stable at temperatures higher than 40°C (Miguez et al., 1993; Guzik et al., 2011); for instance, Wang et al. (2001) reported a C12DO that lost activity after incubation at 45°C for 10 min. Nevertheless, in the thermophilic bacterium *Geobacillus*, the C12DO optimum temperature was 60°C, and it retained 22% activity at 80°C (Giedraityte and Kalėdienė, 2009). Guzik et al. (2011) reported C12DO from *P. putida* with high activity at 35°C, but its activity was reduced at 50°C. In contrast, our enzyme was stable at 45°C and still active after incubation at 50°C for 30 min (Figures 5B,C). Similar to these results, an enzyme from *Candida tropicalis* was relatively stable after 1 h incubation at 50°C (Long et al., 2016).

### Kinetic Parameters, Effects of Ions and Substrate Specificity of PsC12DO

The kinetic analysis of PsC12DO showed *K*_*m*_ and *k*_*cat*_ values (13.2 ± 2.95 μM and 16.13 ± 0.81 s^–1^) comparable to those found in the literature (Figure 6C). The Michaelis constants for catechol obtained for other purified C12DOs range from 1.1 to 67 μM, and the *K*_*m*_ value of the enzyme from *P. stutzeri* GOM2 was similar to those obtained from *P. arvilla*, *Acinetobacter baylyi* ADP, and *Acinetobacter* sp. Y64 (Lin and Milase, 2015; Nazmi et al., 2019). In terms of the catalytic constant *k*_*cat*_, PsC12DO has a moderate value compared to those of other dioxygenases, whose values range from 1.17 to 46 s^–1^. A similar result was observed when comparing the *k*_*cat*_/*K*_*m*_ parameters (1.2 × 10^6^ M^–1^ s^–1^), which reflect a higher catalytic proficiency of PsC12DO than those from *Acinetobacter* sp. Y64, *Vitreoscilla* sp. and *Geobacillus* sp. (Giedraityte and Kalėdienė, 2009; Lin and Milase, 2015; Xu et al., 2017) but a lower proficiency than dioxygenases from *Acinetobacter radioresistens* LGM S13, *Rhodococcus rhodochrous* NCIMB 13259 and *Rhodococcus opacus* 1CP (Caglio et al., 2009; Subbotina et al., 2016).

Catechol 1,2-dioxygenases are active almost exclusively on catechol (Pemberton, 1983). However, a wide range of substrates are being tested. A C12DO from *S. maltophilia* showed activity against 3- and 4-MC but was not active in the presence of chlorocatechols (Guzik et al., 2014). In *Acinetobacter* sp., C12DO was capable of using 4-MC (61%), 4-nitrocatechol (80%) and 1,2,4-benzenetriol (62%) but not 3-MC (Lin and Milase, 2015). Pandeeti and Siddavattam (2011) reported a different *Acinetobacter* sp. strain that showed very strict substrate specificity. Following this line of evidence, our enzyme had low activity on catechol derivatives, including a chlorinated compound, showing 37% relative activity on 4-MC and minor activities (<10%) on 3-MC and 4-CC (Figure 6B).

Many catechol dioxygenases are sensitive to Fe^2+^, Cu^2+^, Mg^2+^, Co^2+^, Mn^2+^, and Zn^2+^ (Matsumura et al., 2004; Wang et al., 2006). PsC12DO was sensitive to Hg^2+^, Zn^2+^, and Fe^3+^ ions; however, we did not observe a critical inactivation when the enzyme was incubated with EDTA or Ca^2+^ (Table 3). EDTA removes the metal at the active site of dioxygenases, and Ca^+^ ions compete with Fe^2+^ and Fe^3+^ at the metal binding site (Sanakis et al., 2003), which might cause alterations in the enzyme structure leading to a partial or total loss of enzyme activity. However, a few reports indicate that ions such as Pb^2+^, Mg^2+^, K^+^, Fe^3+^, Hg^2+^, and Ca^2+^ could enhance the activity of catechol dioxygenases (Wang et al., 2006; Guo et al., 2009); nevertheless, we did not find that any of these could enhance the activity of this enzyme. In contrast to the results obtained by Karamitsu (1968), Ashton and Siegel (1997) and, Silva et al. (2013), where Hg^2+^ promoted the activity of catechol dioxygenases and other enzymes, we observed a total loss of PsC12DO activity, and this negative effect was also reported by Murakami et al. (1998) and Matsumura et al. (2004). There are several reports of low storage stability for C12DOs at 4°C after enzyme purification. Wang et al. (2001) and Guzik et al. (2009) reported that C12DOs from the BA-degrading bacteria *Ralstonia* and *S. maltophilia*, respectively, were inactivated after 2 weeks. Some others C12DOs become inactive after days or weeks of being purified and stored (Strachan et al., 1998). On the other hand, *Geobacillus* sp. C12DO was still active after 2 months (Giedraityte and Kalėdienė, 2009). Our enzyme retained 61% of activity after 6 weeks, suggesting a good stability at low temperatures (Figure 6A), which might be because *P. stutzeri* GOM2 was isolated at a depth of 1,000 m in the sea, where the temperatures are low (2–4°C). We could speculate that the enzyme is adapted to this temperature, even when the optimal temperature for the *Pseudomonas* genus is reported to be 30°C and the optimal temperature for the activity of PsC12DO was determined to be 40°C.

### Molecular Mass of PsC12DO and the Effects of Salinity on Quaternary Structure

There are no previous reports of C12DOs tested in saline conditions; however, because our sample was taken from the swGoM, we determined that the enzyme was still active (60%) at 500 mM NaCl (Figure 7A), making this the first report of a dioxygenase able to retain activity under these conditions *in vitro*. To date, all C12DO three-dimensional structures from Gram-negative bacteria are dimers (Vetting and Ohlendorf, 2000; Earhart et al., 2005), but some other oligomeric states in solution have been reported, such as C12DO from *P. aeruginosa* TKU002 in a monomeric state (22 kDa) (Wang et al., 2006), and a trimeric C12DO was reported in other Gram-negative bacteria from the genus *Acinetobacter* (Briganti et al., 2000). PsC12DO is the first C12DO reported as a trimer in *Pseudomonas* genus, with a molecular mass of 107 kDa. In other bacteria of the same genus, such as *P. arvilla C-1*, both C12DO isozymes (Iso α and Iso β) have been described as (*homo- or hetero-*) dimers (Earhart et al., 2005); this has also been reported in *P. fluorescens* (Saxena and Thakur, 2005). In *A. radioresistens*, the two isozymes of C12DO (Iso A and Iso B) are homodimers, but in conditions of low ionic strength, IsoA can aggregate as a trimer, in contrast to IsoB, which maintains the dimeric structure (Briganti et al., 2000). This effect occurs in PsC12DO where, by changing the ionic strength conditions (50 mM glycine-NaOH buffer, pH 8.5 supplemented with 700 mM NaCl), PsC12DO changes to a dimeric arrangement and causes a 51% decrease in activity.

In addition, the alignment analysis shows that the core of the PsC12DO enzyme is highly conserved, with some variable regions in the N- and C-terminus sequences. In the N-terminus, these differences are located in residues 6–29 (Figure 8). This domain is important to the oligomeric arrangement in C12DOs, where the helices of two monomeric subunits form the hydrophobic cavity at the dimeric interface that contains two lipid molecules (Earhart et al., 2005). In the C-terminus, variable regions are in residues 265–282 and 292–304, and these regions consist mostly of random coils with poorly defined secondary structures located in the vicinity of the active site of the enzyme (Caglio et al., 2009; Nazmi et al., 2019). Changes in both domains in the N- and C-termini could be responsible for the changes in quaternary structure in PsC12DO and its activity. To clarify the relationship between structure-function and ionic force in PsC12DO, we are actively working on crystallographic and structural assays.

The ranges of activity observed in the characterization of our enzyme are comparable with those other C12DO reported as dimers, however; since no other trimer structure has been biochemically characterized, we can only compare the activity of PsC12DO with the ones purified as dimers, but we do not know if it is possible for other enzymes to act as trimers keeping the same properties, or if they remain active or not. In the case of *A. radioresistens* our results are similar to both isoenzymes when compared to the data obtained on pH and temperature, however; they report a decrease in activity in the alkaline pH range (NaOH-glycine) for IsoB, which maintains the dimeric structure. Our trimer is active in alkaline conditions, we do not know if is still active under the conditions that stimulate the dimer conformation, however, we find it interesting to enquire about it.

## Conclusion

Considering that *P. stutzeri* GOM2 strain has only one copy of *catA* gene (other isozymes of C12DOs and genes for C23DOs are absent) in comparison with other bacteria, the presence of a unique PsC12DO and its capacity to change in quaternary structure could be related with the variation in N- and C- termini, and probably are the responsible for the enzyme to be active in a trimeric state. Additionally to the trimeric state, our results showed a broad range of action for PsC12DO in solutions with pH values ranging from neutral to alkaline (7.5–11.5), considering that *P. stutzeri* is not a thermophilic bacteria the optimal temperature is 40°C and still active after incubation at 50°C for 30 min. Including that our enzyme retained 61% of activity after 6 weeks at 4°C, suggesting a good stability at low temperatures and permit a long term storage. And focusing in activity in high salinity concentrations with a 50% of residual activity still present at 500 mM NaCl and the fact that the presence of EDTA or Ca^2+^ did not caused inactivation of PsC12DO, these properties let use this enzyme in environments in presence of EDTA, metals and salinity conditions. We consider that our enzyme has important features that could also be improved by an immobilization technique.

In conclusion, we isolated a *P. stutzeri* strain from seawater containing the whole benzoate degradation pathway that produces a biologically functional C12DO. We characterized this dioxygenase, identifying a trimeric state and its kinetic and biochemical features. We also observed changes in PsC12DO quaternary structure in high salt concentrations (700 mM NaCl), causing a decrease in activity. Furthermore, due to the importance of dioxygenases in bioremediation processes and to take advantage of structural changes and the maintenance of activity of PsC12DO, this enzyme could be useful in bioremediation processes to remove catechols from waste waters and other environments.

## Data Availability Statement

The datasets generated for this study can be found in the submission SUB6629606, “*Pseudomonas stutzeri* GOM2,” accession number WOUM00000000. For WGS genomes the contig accession numbers are present as a hyperlink at the bottom of the WGS master record WOUM00000000.

## Author Contributions

JR-S, AA-J, LM-M, JR-M, ER-P, and LP-L contributed to the design and implementation of the research. EG-L and NR-G performed the computational and bioinformatic analysis. JR-S, AA-J, SM-L, KO-O, and ER-C performed the experiments and analyzed the data. All authors contributed to the writing of the manuscript and approved the final version. LP-L coordinated the IBt-L4-CIGoM group.

## Conflict of Interest

The authors declare that the research was conducted in the absence of any commercial or financial relationships that could be construed as a potential conflict of interest.

## Footnotes

1 http://trinotate.github.io

## Supplementary Material

FIGURE S1 PsC12DO changes in quaternary structure. **(A)** Hydrodynamic diameter (nm) of PsC12DO in 50 mM glycine-NaOH, pH 8.5, showing a value of 10 nm. **(B)** Hydrodynamic diameter of PsC12DO in 50 mM glycine-NaOH, pH 8.5 + 700 mM NaCl, showing a value of 8 nm. The protein concentration in each assay was 1 μg/μL in a 1-mL volume, at 40°C.

FIGURE S2 Acrylamide gel electrophoresis under non-denaturing conditions 10%. 1-KatG *Mycobacterium tuberculosis −80 kDa* (*monomer*) and *160 kDa* (*dimer*). 2-PsC12D samples (*dimer and trimer*) the two bands dimer and trimer are observed (arrows).

TABLE S1 Genomes used for ANI analysis.

TABLE S2 Sequences used to build the phylogenetic tree of dioxygenases.

TABLE S3 Enzymes identified using a set of enzymes involved in the degradation of xenobiotics.

TABLE S4 Taxonomic annotation.

TABLE S5 Location of the enzymes identified in the genome of *P. stutzeri* GOM2.
