# Supplementary figures and images for: Characterization of a Novel Functional Trimeric Catechol 1,2-Dioxygenase From a Pseudomonas stutzeri Isolated From the Gulf of Mexico
Source: Front Microbiol. 2020 Jun 4;11:1100. doi: 10.3389/fmicb.2020.01100 (PMC7287156; doi:10.3389/fmicb.2020.01100)

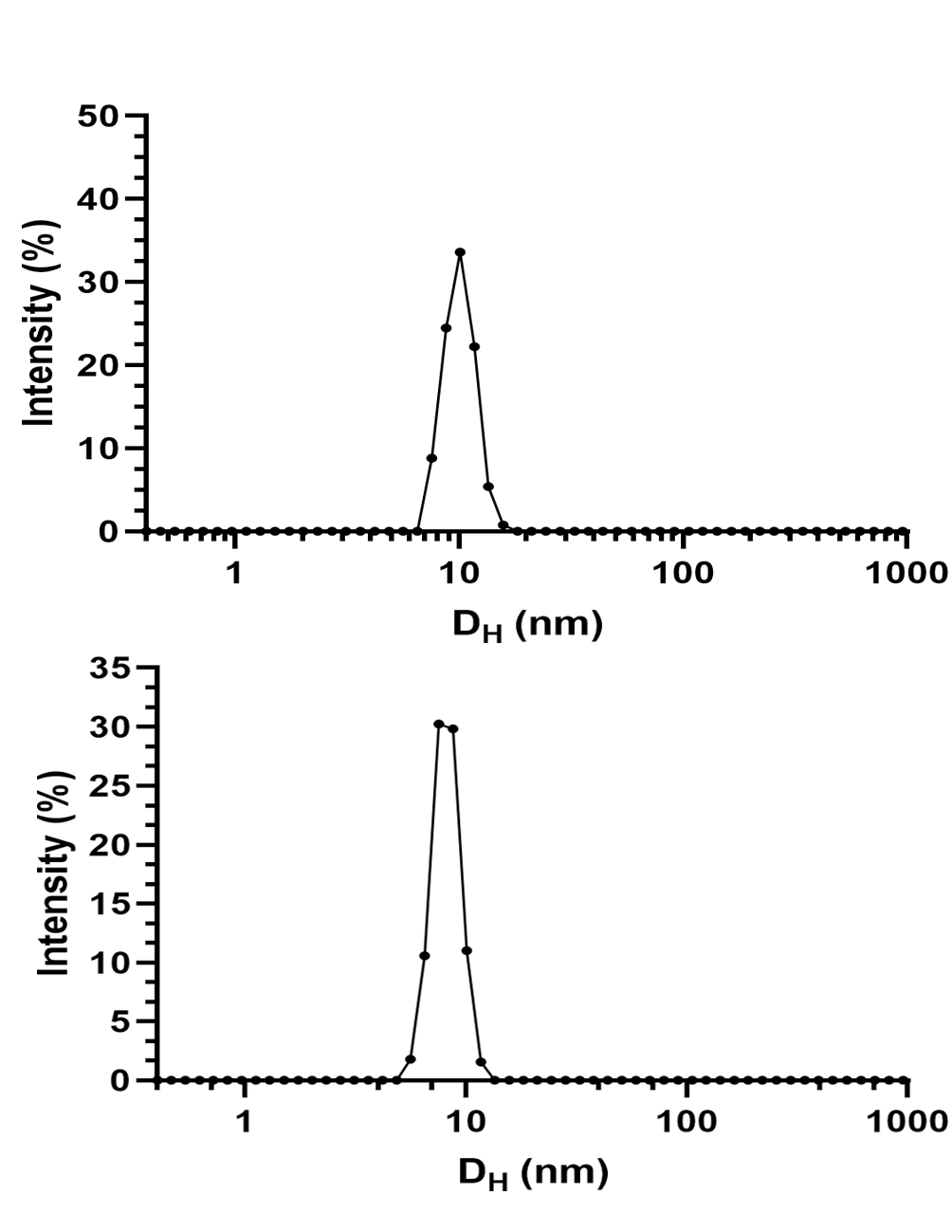

Supplement: FIGURE S1 — PsC12DO changes in quaternary structure. (A) Hydrodynamic diameter (nm) of PsC12DO in 50 mM glycine-NaOH, pH 8.5, showing a value of 10 nm. (B) Hydrodynamic diameter of PsC12DO in 50 mM glycine-NaOH, pH 8.5 + 700 mM NaCl, showing a value of 8 nm. The protein concentration in each assay was 1 μg/μL in a 1-mL volume, at 40°C. [file Image_1.tif]

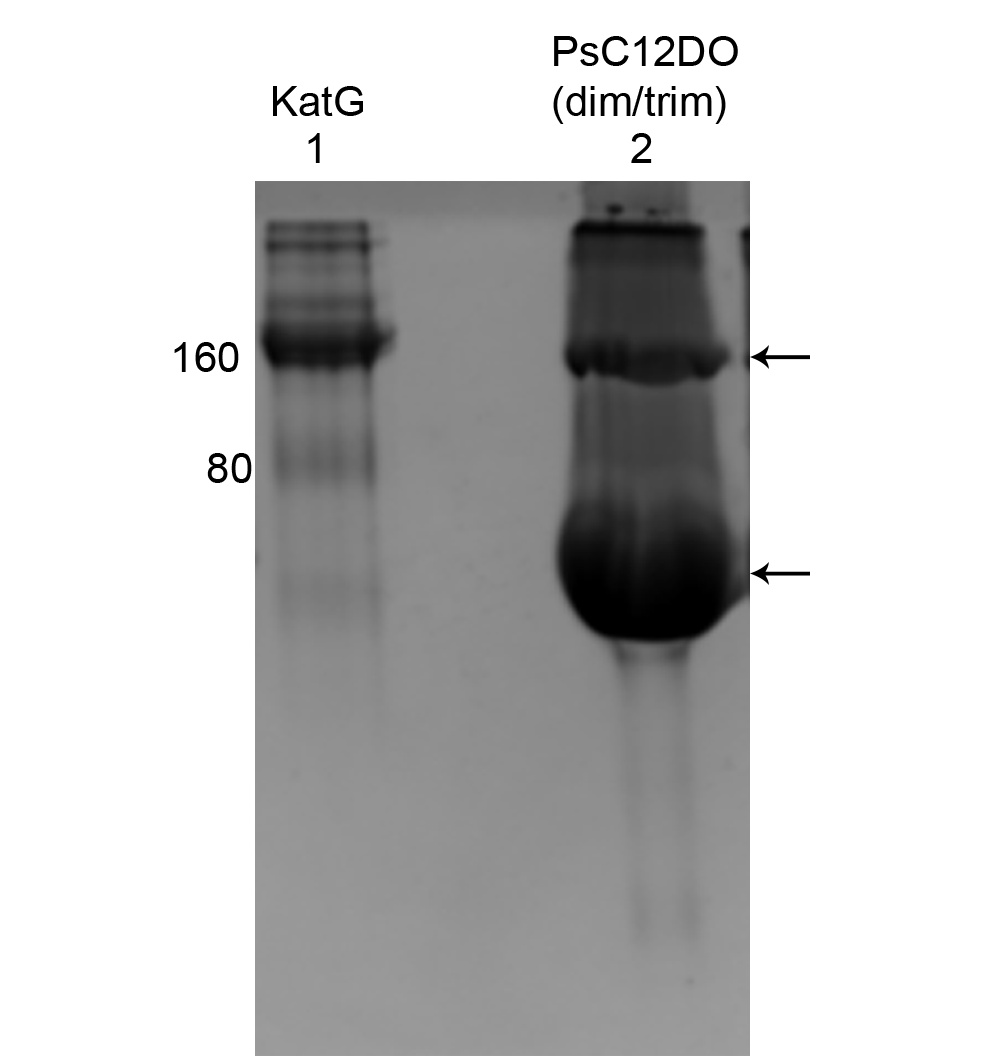

Supplement: FIGURE S2 — Acrylamide gel electrophoresis under non-denaturing conditions 10%. 1-KatG Mycobacterium tuberculosis −80 kDa (monomer) and 160 kDa (dimer). 2-PsC12D samples (dimer and trimer) the two bands dimer and trimer are observed (arrows). [file Image_2.TIFF]
