# Supplementary material for: Characterization of a Novel Functional Trimeric Catechol 1,2-Dioxygenase From a Pseudomonas stutzeri Isolated From the Gulf of Mexico
Source: Front Microbiol. 2020 Jun 4;11:1100. doi: 10.3389/fmicb.2020.01100 (PMC7287156; doi:10.3389/fmicb.2020.01100)
Supplement: TABLE S1 — Genomes used for ANI analysis. [file Data_Sheet_1.docx]

**Supplementary Table 1**

| **Genbank assembly accession** | **Strain** |
| --- | --- |
| GCF_003205815.1_ASM320581v1 | P. sp. R2A2 |
| GCF_005844005.1_ASM584400v1 | P. stutzeri PheN2 |
| GCA_901420285.1_33962_A02 | P. stutzeri NCTC11607 |
| GCF_004793985.1_ASM479398v1 | P. stutzeri CM14 |
| GCF_004683755.1_ASM468375v1 | P. stutzeri AK6 |
| GCF_004359495.1_ASM435949v1 | P. stutzeri ATCC 17588 |
| GCF_900638035.1_55147_C01 | P. stutzeri NCTC10475 |
| GCF_900636845.1_44858_G01 | P. stutzeri NCTC10450 |
| GCF_003965105.1_ASM396510v1 | P. stutzeri PS_001 |
| GCF_003952685.1_ASM395268v1 | P. stutzeri KGS-8 |
| GCF_003952645.1_ASM395264v1 | P. stutzeri KGS-2 |
| GCF_003047145.2_ASM304714v2 | P. stutzeri SGAir0442 |
| GCF_003940985.1_ASM394098v1 | P. stutzeri PS_050 |
| GCF_003940545.1_ASM394054v1 | P. stutzeri PS_066 |
| GCF_003940515.1_ASM394051v1 | P. stutzeri PS_087 |
| GCF_003940495.1_ASM394049v1 | P. stutzeri PS_133 |
| GCF_003936745.1_ASM393674v1 | P. stutzeri PS_075 |
| GCF_003936675.1_ASM393667v1 | P. stutzeri PS_234 |
| GCF_003936705.1_ASM393670v1 | P. stutzeri PS_131 |
| GCF_003936715.1_ASM393671v1 | P. stutzeri PS_110 |
| GCF_003936145.1_ASM393614v1 | P. stutzeri PS_027 |
| GCF_003936125.1_ASM393612v1 | P. stutzeri PS_072 |
| GCF_003936075.1_ASM393607v1 | P. stutzeri PS_125 |
| GCF_003936085.1_ASM393608v1 | P. stutzeri PS_128 |
| GCF_003936055.1_ASM393605v1 | P. stutzeri PS_134 |
| GCF_003936035.1_ASM393603v1 | P. stutzeri PS_130 |
| GCF_003935935.1_ASM393593v1 | P. stutzeri PS_197 |
| GCF_003936025.1_ASM393602v1 | P. stutzeri PS_138 |
| GCF_003935965.1_ASM393596v1 | P. stutzeri PS_151 |
| GCF_003935955.1_ASM393595v1 | P. stutzeri PS_211 |
| GCF_003935925.1_ASM393592v1 | P. stutzeri PS_167 |
| GCF_003935625.1_ASM393562v1 | P. stutzeri PS_377 |
| GCF_003935565.1_ASM393556v1 | P. stutzeri PS_257 |
| GCF_003935575.1_ASM393557v1 | P. stutzeri PS_366 |
| GCF_003935545.1_ASM393554v1 | P. stutzeri PS_376 |
| GCA_003410115.1_ASM341011v1 | P. stutzeri ATCC 17587 |
| GCF_900455625.1_57940_C02 | P. stutzeri NCTC10473 |
| GCF_003255915.1_ASM325591v1 | P. stutzeri CM |
| GCA_003243385.1_ASM324338v1 | P. stutzeri S2_003_000_R2_15 |
| GCF_003047145.1_ASM304714v1 | P. stutzeri SGAir0442 |
| GCF_003008495.1_ASM300849v1 | P. stutzeri 1W1-1A |
| GCF_003001655.1_ASM300165v1 | P. stutzeri DW2-1 |
| GCF_002909485.1_ASM290948v1 | P. stutzeri 24a13 |
| GCF_002891015.1_ASM289101v1 | P. stutzeri DSM 50238 |
| GCF_002890995.1_ASM289099v1 | P. stutzeri 28a3 |
| GCF_002890955.1_ASM289095v1 | P. stutzeri ST27MN3 |
| GCF_002890935.1_ASM289093v1 | P. stutzeri DNSP21 |
| GCF_002890915.1_ASM289091v1 | P. stutzeri 24a75 |
| GCF_002890895.1_ASM289089v1 | P. stutzeri 4C29 |
| GCF_002890875.1_ASM289087v1 | P. stutzeri DSM 17088 |
| GCF_002890855.1_ASM289085v1 | P. stutzeri CCUG 36651 |
| GCF_002890835.1_ASM289083v1 | P. stutzeri CCUG 44592 |
| GCF_002890795.1_ASM289079v1 | P. stutzeri KC |
| GCF_002843895.1_ASM284389v1 | P. stutzeri DSM 50227 |
| GCA_002501025.1_ASM250102v1 | P. stutzeri UBA7897 |
| GCA_002500775.1_ASM250077v1 | P. stutzeri UBA7844 |
| GCA_002500675.1_ASM250067v1 | P. stutzeri UBA7861 |
| GCA_002500285.1_ASM250028v1 | P. stutzeri UBA7847 |
| GCA_002487265.1_ASM248726v1 | P. stutzeri UBA4963 |
| GCA_002484325.1_ASM248432v1 | P. stutzeri UBA7613 |
| GCA_002484245.1_ASM248424v1 | P. stutzeri UBA7617 |
| GCA_002484035.1_ASM248403v1 | P. stutzeri UBA7627 |
| GCA_002483925.1_ASM248392v1 | P. stutzeri UBA7633 |
| GCA_002483545.1_ASM248354v1 | P. stutzeri UBA7652 |
| GCA_002482365.1_ASM248236v1 | P. stutzeri UBA7711 |
| GCA_002482335.1_ASM248233v1 | P. stutzeri UBA7713 |
| GCA_002479445.1_ASM247944v1 | P. stutzeri UBA7511 |
| GCA_002479285.1_ASM247928v1 | P. stutzeri UBA7538 |
| GCA_002477745.1_ASM247774v1 | P. stutzeri UBA7601 |
| GCA_002477385.1_ASM247738v1 | P. stutzeri UBA7458 |
| GCA_002476945.1_ASM247694v1 | P. stutzeri UBA7516 |
| GCA_002473225.1_ASM247322v1 | P. stutzeri UBA7241 |
| GCA_002472825.1_ASM247282v1 | P. stutzeri UBA7250 |
| GCA_002471085.1_ASM247108v1 | P. stutzeri UBA7343 |
| GCA_002455935.1_ASM245593v1 | P. stutzeri UBA6631 |
| GCA_002455815.1_ASM245581v1 | P. stutzeri UBA6635 |
| GCA_002455775.1_ASM245577v1 | P. stutzeri UBA6639 |
| GCA_002455715.1_ASM245571v1 | P. stutzeri UBA6643 |
| GCA_002455635.1_ASM245563v1 | P. stutzeri UBA6649 |
| GCA_002454635.1_ASM245463v1 | P. stutzeri UBA6702 |
| GCA_002453575.1_ASM245357v1 | P. stutzeri UBA6752 |
| GCA_002453425.1_ASM245342v1 | P. stutzeri UBA6760 |
| GCA_002452485.1_ASM245248v1 | P. stutzeri UBA6822 |
| GCA_002451325.1_ASM245132v1 | P. stutzeri UBA6892 |
| GCA_002451245.1_ASM245124v1 | P. stutzeri UBA6896 |
| GCA_002449475.1_ASM244947v1 | P. stutzeri UBA6826 |
| GCA_002440565.1_ASM244056v1 | P. stutzeri UBA6275 |
| GCA_002440495.1_ASM244049v1 | P. stutzeri UBA6287 |
| GCA_002439525.1_ASM243952v1 | P. stutzeri UBA6279 |
| GCA_002439185.1_ASM243918v1 | P. stutzeri UBA6312 |
| GCA_002438285.1_ASM243828v1 | P. stutzeri UBA6301 |
| GCA_002438105.1_ASM243810v1 | P. stutzeri UBA6318 |
| GCA_002435225.1_ASM243522v1 | P. stutzeri UBA6501 |
| GCA_002434545.1_ASM243454v1 | P. stutzeri UBA6548 |
| GCA_002434415.1_ASM243441v1 | P. stutzeri UBA6556 |
| GCA_002434325.1_ASM243432v1 | P. stutzeri UBA6559 |
| GCA_002433525.1_ASM243352v1 | P. stutzeri UBA6599 |
| GCA_002432785.1_ASM243278v1 | P. stutzeri UBA5840 |
| GCA_002432185.1_ASM243218v1 | P. stutzeri UBA5863 |
| GCA_002432145.1_ASM243214v1 | P. stutzeri UBA5865 |
| GCA_002430985.1_ASM243098v1 | P. stutzeri UBA5929 |
| GCA_002430265.1_ASM243026v1 | P. stutzeri UBA5963 |
| GCA_002429975.1_ASM242997v1 | P. stutzeri UBA5981 |
| GCA_002429895.1_ASM242989v1 | P. stutzeri UBA5985 |
| GCA_002425655.1_ASM242565v1 | P. stutzeri UBA5562 |
| GCA_002425495.1_ASM242549v1 | P. stutzeri UBA5569 |
| GCA_002424945.1_ASM242494v1 | P. stutzeri UBA5597 |
| GCA_002423335.1_ASM242333v1 | P. stutzeri UBA6125 |
| GCA_002421085.1_ASM242108v1 | P. stutzeri UBA5649 |
| GCA_002420805.1_ASM242080v1 | P. stutzeri UBA5661 |
| GCA_002419865.1_ASM241986v1 | P. stutzeri UBA5710 |
| GCA_002419855.1_ASM241985v1 | P. stutzeri UBA5711 |
| GCA_002419685.1_ASM241968v1 | P. stutzeri UBA5719 |
| GCA_002418345.1_ASM241834v1 | P. stutzeri UBA5790 |
| GCA_002415415.1_ASM241541v1 | P. stutzeri UBA5140 |
| GCA_002415305.1_ASM241530v1 | P. stutzeri UBA5029 |
| GCA_002414315.1_ASM241431v1 | P. stutzeri UBA5117 |
| GCA_002413985.1_ASM241398v1 | P. stutzeri UBA5137 |
| GCA_002403755.1_ASM240375v1 | P. stutzeri UBA4749 |
| GCA_002403535.1_ASM240353v1 | P. stutzeri UBA4758 |
| GCA_002392215.1_ASM239221v1 | P. stutzeri UBA3873 |
| GCA_002392165.1_ASM239216v1 | P. stutzeri UBA3876 |
| GCA_002392095.1_ASM239209v1 | P. stutzeri UBA3879 |
| GCA_002392045.1_ASM239204v1 | P. stutzeri UBA3882 |
| GCA_002391985.1_ASM239198v1 | P. stutzeri UBA3885 |
| GCA_002389895.1_ASM238989v1 | P. stutzeri UBA4391 |
| GCA_002389165.1_ASM238916v1 | P. stutzeri UBA4480 |
| GCA_002389115.1_ASM238911v1 | P. stutzeri UBA4482 |
| GCA_002388215.1_ASM238821v1 | P. stutzeri UBA4529 |
| GCA_002388145.1_ASM238814v1 | P. stutzeri UBA4533 |
| GCA_002388065.1_ASM238806v1 | P. stutzeri UBA4537 |
| GCA_002387945.1_ASM238794v1 | P. stutzeri UBA4545 |
| GCA_002387495.1_ASM238749v1 | P. stutzeri UBA4595 |
| GCA_002387375.1_ASM238737v1 | P. stutzeri UBA4614 |
| GCA_002387305.1_ASM238730v1 | P. stutzeri UBA4547 |
| GCA_002387205.1_ASM238720v1 | P. stutzeri UBA4556 |
| GCA_002387165.1_ASM238716v1 | P. stutzeri UBA4558 |
| GCA_002386665.1_ASM238666v1 | P. stutzeri UBA4603 |
| GCA_002385235.1_ASM238523v1 | P. stutzeri UBA3963 |
| GCA_002385035.1_ASM238503v1 | P. stutzeri UBA3971 |
| GCA_002384505.1_ASM238450v1 | P. stutzeri UBA3984 |
| GCA_002381725.1_ASM238172v1 | P. stutzeri UBA4107 |
| GCA_002381675.1_ASM238167v1 | P. stutzeri UBA4113 |
| GCA_002380885.1_ASM238088v1 | P. stutzeri UBA4134 |
| GCA_002380585.1_ASM238058v1 | P. stutzeri UBA4160 |
| GCA_002380045.1_ASM238004v1 | P. stutzeri UBA4155 |
| GCA_002379995.1_ASM237999v1 | P. stutzeri UBA4163 |
| GCA_002378825.1_ASM237882v1 | P. stutzeri UBA3453 |
| GCA_002378715.1_ASM237871v1 | P. stutzeri UBA3460 |
| GCA_002378365.1_ASM237836v1 | P. stutzeri UBA3454 |
| GCA_002377205.1_ASM237720v1 | P. stutzeri UBA3517 |
| GCA_002376625.1_ASM237662v1 | P. stutzeri UBA3552 |
| GCA_002375735.1_ASM237573v1 | P. stutzeri UBA3589 |
| GCA_002367555.1_ASM236755v1 | P. stutzeri UBA3044 |
| GCA_002365745.1_ASM236574v1 | P. stutzeri UBA3094 |
| GCA_002365475.1_ASM236547v1 | P. stutzeri UBA3114 |
| GCA_002365145.1_ASM236514v1 | P. stutzeri UBA3137 |
| GCA_002365105.1_ASM236510v1 | P. stutzeri UBA3140 |
| GCA_002365055.1_ASM236505v1 | P. stutzeri UBA3146 |
| GCA_002363465.1_ASM236346v1 | P. stutzeri UBA3230 |
| GCA_002363305.1_ASM236330v1 | P. stutzeri UBA3248 |
| GCA_002363025.1_ASM236302v1 | P. stutzeri UBA3228 |
| GCA_002362915.1_ASM236291v1 | P. stutzeri UBA3235 |
| GCA_002362885.1_ASM236288v1 | P. stutzeri UBA3232 |
| GCA_002362825.1_ASM236282v1 | P. stutzeri UBA3240 |
| GCA_002362755.1_ASM236275v1 | P. stutzeri UBA3244 |
| GCA_002360755.1_ASM236075v1 | P. stutzeri UBA3349 |
| GCA_002359175.1_ASM235917v1 | P. stutzeri UBA2672 |
| GCA_002358225.1_ASM235822v1 | P. stutzeri UBA3423 |
| GCA_002358195.1_ASM235819v1 | P. stutzeri UBA3418 |
| GCA_002354345.1_ASM235434v1 | P. stutzeri UBA2699 |
| GCA_002354255.1_ASM235425v1 | P. stutzeri UBA2703 |
| GCA_002352925.1_ASM235292v1 | P. stutzeri UBA1884 |
| GCA_002348875.1_ASM234887v1 | P. stutzeri UBA2949 |
| GCA_002346755.1_ASM234675v1 | P. stutzeri UBA3023 |
| GCA_002346465.1_ASM234646v1 | P. stutzeri UBA3004 |
| GCA_002345575.1_ASM234557v1 | P. stutzeri UBA2301 |
| GCA_002345265.1_ASM234526v1 | P. stutzeri UBA2314 |
| GCA_002345055.1_ASM234505v1 | P. stutzeri UBA2326 |
| GCA_002342105.1_ASM234210v1 | P. stutzeri UBA2472 |
| GCA_002341925.1_ASM234192v1 | P. stutzeri UBA2482 |
| GCA_002341735.1_ASM234173v1 | P. stutzeri UBA2490 |
| GCA_002341565.1_ASM234156v1 | P. stutzeri UBA2499 |
| GCA_002340215.1_ASM234021v1 | P. stutzeri UBA2568 |
| GCA_002340005.1_ASM234000v1 | P. stutzeri UBA2605 |
| GCA_002339875.1_ASM233987v1 | P. stutzeri UBA2573 |
| GCA_002339845.1_ASM233984v1 | P. stutzeri UBA2580 |
| GCA_002339675.1_ASM233967v1 | P. stutzeri UBA2609 |
| GCA_002338865.1_ASM233886v1 | P. stutzeri UBA1830 |
| GCA_002338305.1_ASM233830v1 | P. stutzeri UBA1862 |
| GCA_002338285.1_ASM233828v1 | P. stutzeri UBA1864 |
| GCA_002337885.1_ASM233788v1 | P. stutzeri UBA1890 |
| GCA_002337855.1_ASM233785v1 | P. stutzeri UBA1892 |
| GCA_002337845.1_ASM233784v1 | P. stutzeri UBA1891 |
| GCA_002337815.1_ASM233781v1 | P. stutzeri UBA1893 |
| GCA_002337765.1_ASM233776v1 | P. stutzeri UBA1896 |
| GCA_002337335.1_ASM233733v1 | P. stutzeri UBA1920 |
| GCA_002335105.1_ASM233510v1 | P. stutzeri UBA2003 |
| GCA_002333255.1_ASM233325v1 | P. stutzeri UBA2056 |
| GCA_002332925.1_ASM233292v1 | P. stutzeri UBA2054 |
| GCA_002332525.1_ASM233252v1 | P. stutzeri UBA2064 |
| GCA_002331665.1_ASM233166v1 | P. stutzeri UBA2081 |
| GCA_002331645.1_ASM233164v1 | P. stutzeri UBA2080 |
| GCA_002331415.1_ASM233141v1 | P. stutzeri UBA2115 |
| GCA_002325885.1_ASM232588v1 | P. stutzeri UBA1453 |
| GCA_002325895.1_ASM232589v1 | P. stutzeri UBA1452 |
| GCA_002325835.1_ASM232583v1 | P. stutzeri UBA1455 |
| GCA_002325585.1_ASM232558v1 | P. stutzeri UBA1468 |
| GCA_002325525.1_ASM232552v1 | P. stutzeri UBA1470 |
| GCA_002325025.1_ASM232502v1 | P. stutzeri UBA1495 |
| GCA_002323655.1_ASM232365v1 | P. stutzeri UBA1502 |
| GCA_002323015.1_ASM232301v1 | P. stutzeri UBA1642 |
| GCA_002322705.1_ASM232270v1 | P. stutzeri UBA1598 |
| GCA_002321985.1_ASM232198v1 | P. stutzeri UBA1599 |
| GCA_002321815.1_ASM232181v1 | P. stutzeri UBA1637 |
| GCA_002321095.1_ASM232109v1 | P. stutzeri UBA1646 |
| GCA_002321055.1_ASM232105v1 | P. stutzeri UBA1648 |
| GCA_002320895.1_ASM232089v1 | P. stutzeri UBA1656 |
| GCA_002315125.1_ASM231512v1 | P. stutzeri UBA1804 |
| GCA_002315115.1_ASM231511v1 | P. stutzeri UBA1810 |
| GCA_002314405.1_ASM231440v1 | P. stutzeri UBA1807 |
| GCA_002311755.1_ASM231175v1 | P. stutzeri UBA1168 |
| GCA_002311715.1_ASM231171v1 | P. stutzeri UBA1171 |
| GCA_002311095.1_ASM231109v1 | P. stutzeri UBA1169 |
| GCA_002307985.1_ASM230798v1 | P. stutzeri UBA1286 |
| GCA_002307875.1_ASM230787v1 | P. stutzeri UBA1292 |
| GCA_002307775.1_ASM230777v1 | P. stutzeri UBA1300 |
| GCA_002306155.1_ASM230615v1 | P. stutzeri UBA1355 |
| GCA_002306135.1_ASM230613v1 | P. stutzeri UBA1356 |
| GCA_002296595.1_ASM229659v1 | P. stutzeri UBA808 |
| GCA_002296385.1_ASM229638v1 | P. stutzeri UBA803 |
| GCA_002296325.1_ASM229632v1 | P. stutzeri UBA805 |
| GCA_002296205.1_ASM229620v1 | P. stutzeri UBA821 |
| GCA_002295785.1_ASM229578v1 | P. stutzeri UBA861 |
| GCA_002295365.1_ASM229536v1 | P. stutzeri UBA863 |
| GCA_002295205.1_ASM229520v1 | P. stutzeri UBA872 |
| GCA_002294535.1_ASM229453v1 | P. stutzeri UBA904 |
| GCA_002294115.1_ASM229411v1 | P. stutzeri UBA897 |
| GCA_002293905.1_ASM229390v1 | P. stutzeri UBA915 |
| GCA_002292125.1_ASM229212v1 | P. stutzeri UBA990 |
| GCA_002292085.1_ASM229208v1 | P. stutzeri UBA988 |
| GCF_002282995.1_ASM228299v1 | P. stutzeri KMS 55 |
| GCF_002193135.1_ASM219313v1 | P. stutzeri 19 |
| GCF_002093045.2_ASM209304v2 | P. stutzeri LH-42 |
| GCF_002093045.1_ASM209304v1 | P. stutzeri LH-42 |
| GCF_002027175.1_ASM202717v1 | P. stutzeri 40D2 |
| GCF_001996325.1_ASM199632v1 | P. stutzeri DCP-Ps1 |
| GCF_001705635.1_ASM170563v1 | P. stutzeri AR9-4 |
| GCF_001648195.1_ASM164819v1 | P. stutzeri 273 |
| GCF_001635435.1_ASM163543v1 | P. stutzeri HI00D01 |
| GCF_001575085.1_ASM157508v1 | P. stutzeri ODKF13 |
| GCF_000282955.1_ASM28295v1 | P. stutzeri T13 |
| GCF_000235745.1_ASM23574v2 | P. stutzeri SDM-LAC |
| GCF_000280555.1_ASM28055v1 | P. stutzeri XLDN-R |
| GCF_000341615.1_PstNF13_1.0 | P. stutzeri NF13 |
| GCF_000327065.1_ASM32706v1 | P. stutzeri RCH2 |
| GCF_001294145.1_ASM129414v1 | P. stutzeri KF716 |
| GCF_000263395.1_ASM26339v1 | P. stutzeri TS44 |
| GCF_001276475.1_ASM127647v1 | P. stutzeri C2 |
| GCF_000237885.1_PseStu_1.0 | P. stutzeri CCUG 16156 |
| GCF_000267545.1_ASM26754v1 | P. stutzeri CCUG 29243 |
| GCF_000279165.1_ASM27916v1 | P. stutzeri DSM 10701 |
| GCF_000195105.1_ASM19510v1 | P. stutzeri DSM 4166 |
| GCF_001064225.1_ASM106422v1 | P. stutzeri 267_PSTU |
| GCF_001062345.1_ASM106234v1 | P. stutzeri 1223_PMEN |
| GCF_001038645.1_ASM103864v1 | P. stutzeri SLG510A3-8 |
| GCF_000982865.1_ASM98286v1 | P. stutzeri ST-9 |
| GCF_000952685.1_ASM95268v1 | P. stutzeri NT0128 |
| GCF_000952205.1_ASM95220v1 | P. stutzeri NT0124 |
| GCF_000935215.1_ASM93521v1 | P. stutzeri BAL361 |
| GCF_000219605.1_ASM21960v1 | P. stutzeri CGMCC 1.1803 |
| GCF_000828205.1_ASM82820v1 | P. stutzeri YC-YH1 |
| GCF_000661915.1_ASM66191v1 | P. stutzeri 19SMN4 |
| GCF_000590475.1_ASM59047v1 | P. stutzeri 28a24 |
| GCF_000307775.2_PseStu2.0 | P. stutzeri KOS6 |
| GCF_000455665.1_ASM45566v1 | P. stutzeri MF28 |
| GCF_000416345.1_PstB1SMN1_1.0 | P. stutzeri B1SMN1 |
| GCF_000013785.1_ASM1378v1 | P. stutzeri A1501 |

**Supplementary Table 2. Sequences used to build the phylogenetic tree of dioxygenases**

| **Sequences obtained through blast** | | |  |
| --- | --- | --- | --- |
| **Database** | **ID sequence match** | **Percent Identity** |  |
| P. stutzeri Vs database nr | HAJ87005.1 | 99.038 |  |
|  | WP_063544000.1 | 98.718 |  |
|  | WP_133455780.1 | 98.718 |  |
|  | WP_125869588.1 | 98.397 |  |
|  | WP_110777513.1 | 98.397 |  |
| *P. stutzeri* Vs nr without *P. stutzeri* | MAL91162.1 | 97.742 |  |
|  | HCL15434.1 | 96.474 |  |
|  | CJL61736.1 | 95.513 |  |
|  | PNF99967.1 | 91.987 |  |
|  | WP_090523539.1 | 91.346 |  |
| *P. stutzer*i GOM2 Vs *P. putida* | WP_084857903.1 | 79.167 |  |
|  | WP_079228244.1 | 76.603 |  |
|  | WP_099593249.1 | 76.603 |  |
|  | WP_075805444.1 | 76.603 |  |
|  | WP_075044942.1 | 76.677 |  |
| *P. stutzeri* vs without taxon Pseudomonas | CJL61736.1 | 95.513 |  |
|  | WP_067383272.1 | 72.903 |  |
|  | WP_067292069.1 | 72.903 |  |
|  | SCZ15571.1 | 75.962 |  |
|  | SSU86403.1 | 76.206 |  |
|  | WP_076724300.1 | 59.164 |  |
|  | MAC21167.1 | 58.71 |  |
|  | WP_022990605.1 | 57.419 |  |
|  | WP_111498031.1 | 59.677 |  |

| **Sequences of the paper Guzik et al 2012** | | |  |  |  |
| --- | --- | --- | --- | --- | --- |
| **ID** | **Database** | **Name** | **Organism** | **Substrate** | **Paper Reference ID** |
| Q93SY8 | Uni-prot | Catechol 1,2-dioxygenase | Acinetobacter radioresistens | catechol | AF380158 |
| Q9F103 | Uni-prot | Catechol 1,2-dioxygenase | Acinetobacter radioresistens | 4-methylcatechol | AF182166 |
| CAA85386.1 | NCBI | catechol 1,2-dioxygenase [Acinetobacter calcoaceticus] | Acinetobacter calcoaceticus | catechol | No ID |
| BAD11154.1 | NCBI | catechol 1,2-dioxygenase [Acinetobacter calcoaceticus] | Arthrobacter sp. BA-5-17 | Catechol, 3-methylcatechol, 4-methylcatechol | AB109791.1 |
| BAD11152.1 | NCBI | cis,cis-muconate cycloisomerase | Arthrobacter sp. BA-5-17 | // | NA |
| BAC16779.1 | NCBI | catechol 1,2-dioxygenase | Burkholderia sp. TH2 | Catechol, 3-methylcatechol, 4-methylcatechol | AB035483 |
| BAC16769.1 | NCBI | catechol 1,2-dioxygenase | Burkholderia sp. TH2 | Catechol, 4-methylcatechol | AB035325 |
| AAG05895.1 | NCBI | catechol 1,2-dioxygenase | Pseudomonas aeruginosa PAO1 |  |  |
| ABS86779.1 | NCBI | catechol 1,2-dioxygenase | Pseudomonas putida | catechol | EU000396.1 |
| WP_084166591.1 | NCBI | catechol 1,2-dioxygenase | Pseudomonas knackmussii | Catechol,3-chlorocatechol, 4-chlorocatechol, 3-methylcatechol, 4-methylcatechol | No ID |
| P96984_RHOER | Uni-prot | catechol 1,2-dioxygenase | Rhodococcus erythropolis | catechol | D83237 |
| CAA67941.1 | NCBI | catechol 1,2-dioxygenase | Rhodococcus opacus | Catechol, 3-methylcatechol, 4-methylcatechol | X99622.1 |
| AAC38251.1 | NCBI | chlorocatechol 1,2-dioxygenase | Rhodococcus opacus | 4-chlorocatecho | AF003948.1 |
| CAD28142.1 | NCBI | chlorocatechol 1,2-dioxygenase | (plasmid) Rhodococcus opacus | 3-chlorocatechol | AJ439407.1 |
| ABS86780.1 | NCBI | catechol 1,2-dioxygenase | Stenotrophomonas maltophilia | catechol |  |

| **Sequences of the paper Nazmi et al. 2019 and Caglio 2009** | | | |
| --- | --- | --- | --- |
| ID | Name | Organism | Reference |
| Q9F103 | CatAb | A. radioresistens | Nazmi et al. 2019-Caglio et al. 2009-Guzik |
| Q51433 | CatA C12DO | P. putida | Nazmi et al. 2019-Caglio et al. 2009 |
| A4JR51 | C12DO | B. vietnamiensis | Nazmi et al. 2019 |
| P07773.1 | C12DO | A. baylyi ASP1 | Nazmi et al. 2019-Caglio et al. 2009 |
| P11451.1 | CC12DO | P. putida | Nazmi et al. 2019-Caglio et al. 2009 |
| O67987 | CC12DO | R. opacus | Nazmi et al. 2019 |
| P95607 | CatA C12DO (Fragment) | R. opacus | Nazmi et al. 2019 |
| O33948 | CATA1, C12DO | Acinetobacter lwoffii | Caglio et al. 2009 |
| Q8G9L3 | clcA2, CC12DO | P. putida | Caglio et al. 2009 |
| O67987 | CLCA | [R. opacus | Caglio et al. 2009 |
| Q9WXC8 | CC12DO | P. putida | Caglio et al. 2009 |
| P27098.1 | CC12DO | Pseudomonas sp. P51 | Caglio et al. 2009 |
| AAQ87516.1 | Hq12DO (plasmid) | S. fredii NGR234 | Caglio et al. 2009 |
| CAA51371.1 | Hq12DO | Sphingomonas sp. | Caglio et al. 2009 |

**Supplementary Table 3**

| **Enzymes identified using a set of enzymes involved in the degradation of xenobiotics** | | | | | |
| --- | --- | --- | --- | --- | --- |
|  | EC-search | Location in genome | Percent identity | |  |
| Scaffold 15 | EC_1.14.12.10 | 1182 | 71.882 |  |  |
|  | EC_1.14.12.10 | 1183 | 61.728 |  |  |
|  | EC_1.3.1.25 | 1185 | 60.153 |  |  |
|  | EC_5.5.1.1 | 1187 | 84.595 |  |  |
|  | EC_5.3.3.4 | 1188 | 85.417 |  |  |
|  | EC_1.13.11.1 | 1189 | 73.718 |  |  |
|  |  |  |  |  |  |
| Scaffold 14 | EC_2.8.3.6 | 1146 | 90.769 |  |  |
|  | EC_2.8.3.6 | 1147 | 87.589 |  |  |
|  | EC_2.3.1.174 | 1145 | 82.045 |  |  |
|  | EC_3.1.1.24 | 1144 | 41.27 |  |  |

| **Supplementary Table 4. Taxonomic annotation** | | | | | |
| --- | --- | --- | --- | --- | --- |
| **Method** | **Software** | **Database** | **Result*** | **Score** | **Taxa level** |
| 16S rRNA | Blast | 16S RefSeq NCBI | *Pseudomonas stutzeri* strain ATCC 17588 (NR_103934) | 99.803 % Identity | Species |
|  |  | Greengenes 13_5 | *Pseudomonas stutzeri* DSM 4166 (CP002622.1) | 99.869 % Identity | Species |
|  |  | Silva SSU 132 | *Pseudomonas stutzeri* B1SMN1 (AMVM00000000.1) | 99.869 % Identity | Species |
|  |  | RDP Release 11, Update 5 | Pseudomonas stutzeri A1501 (CP000304.1) | 99.869 % Identity | Species |
| k-mer | Kraken | Kraken | *Pseudomonas* | 96.64 % sequences | Genus |
| Single Copy Markers | MetaPhlan | MetaPhlan | *Pseudomonas stutzeri* unclassified | 99.3757 % sequences | Species |

**Supplementary Table 5**

| **Scaffold** | **ID** | **Start** | **Stop** | **Strand** | **Function** |
| --- | --- | --- | --- | --- | --- |
| scaffold_14 | [1144](http://rast.nmpdr.org/seedviewer.cgi?page=Annotation&feature=fig%7C6666666.294894.peg.1144) | 20091 | 19303 | - | Beta-ketoadipate enol-lactone hydrolase (EC 3.1.1.24) |
| scaffold_14 | [1145](http://rast.nmpdr.org/seedviewer.cgi?page=Annotation&feature=fig%7C6666666.294894.peg.1145) | 21553 | 20348 | - | Beta-ketoadipyl CoA thiolase (EC 2.3.1.-) |
| scaffold_14 | [1146](http://rast.nmpdr.org/seedviewer.cgi?page=Annotation&feature=fig%7C6666666.294894.peg.1146) | 22332 | 21550 | - | 3-oxoadipate CoA-transferase subunit B (EC 2.8.3.6); Glutaconate CoA-transferase subunit B (EC 2.8.3.12) |
| scaffold_14 | [1147](http://rast.nmpdr.org/seedviewer.cgi?page=Annotation&feature=fig%7C6666666.294894.peg.1147) | 23189 | 22332 | - | 3-oxoadipate CoA-transferase subunit A (EC 2.8.3.6); Glutaconate CoA-transferase subunit A (EC 2.8.3.12) |
| scaffold_14 | [1148](http://rast.nmpdr.org/seedviewer.cgi?page=Annotation&feature=fig%7C6666666.294894.peg.1148) | 23220 | 23387 | + | hypothetical protein |
| scaffold_14 | [1149](http://rast.nmpdr.org/seedviewer.cgi?page=Annotation&feature=fig%7C6666666.294894.peg.1149) | 24207 | 23365 | - | Pca regulon regulatory protein PcaR |
|  |  |  |  |  |  |
| scaffold_15 | [1181](http://rast.nmpdr.org/seedviewer.cgi?page=Annotation&feature=fig%7C6666666.294894.peg.1181) | 30562 | 31515 | + | benABC operon transcriptional activator BenR |
| scaffold_15 | [1182](http://rast.nmpdr.org/seedviewer.cgi?page=Annotation&feature=fig%7C6666666.294894.peg.1182) | 31733 | 33109 | + | Benzoate 1,2-dioxygenase alpha subunit (EC 1.14.12.10) |
| scaffold_15 | [1183](http://rast.nmpdr.org/seedviewer.cgi?page=Annotation&feature=fig%7C6666666.294894.peg.1183) | 33110 | 33598 | + | Benzoate 1,2-dioxygenase beta subunit (EC 1.14.12.10) |
| scaffold_15 | [1184](http://rast.nmpdr.org/seedviewer.cgi?page=Annotation&feature=fig%7C6666666.294894.peg.1184) | 33609 | 34619 | + | benzoate dioxygenase, ferredoxin reductase component |
| scaffold_15 | [1185](http://rast.nmpdr.org/seedviewer.cgi?page=Annotation&feature=fig%7C6666666.294894.peg.1185) | 34690 | 35466 | + | 1,2-dihydroxycyclohexa-3,5-diene-1-carboxylate dehydrogenase (EC 1.3.1.25) |
| scaffold_15 | [1186](http://rast.nmpdr.org/seedviewer.cgi?page=Annotation&feature=fig%7C6666666.294894.peg.1186) | 35574 | 36920 | + | benzoate MFS transporter BenK |
| scaffold_15 | [1187](http://rast.nmpdr.org/seedviewer.cgi?page=Annotation&feature=fig%7C6666666.294894.peg.1187) | 36948 | 38069 | + | Muconate cycloisomerase (EC 5.5.1.1) |
| scaffold_15 | [1188](http://rast.nmpdr.org/seedviewer.cgi?page=Annotation&feature=fig%7C6666666.294894.peg.1188) | 38084 | 38374 | + | Muconolactone isomerase (EC 5.3.3.4) |
| scaffold_15 | [1189](http://rast.nmpdr.org/seedviewer.cgi?page=Annotation&feature=fig%7C6666666.294894.peg.1189) | 38446 | 39384 | + | Catechol 1,2-dioxygenase (EC 1.13.11.1) |
| scaffold_15 | [1190](http://rast.nmpdr.org/seedviewer.cgi?page=Annotation&feature=fig%7C6666666.294894.peg.1190) | 39523 | 40722 | + | Benzoate transport protein |
| scaffold_15 | [1191](http://rast.nmpdr.org/seedviewer.cgi?page=Annotation&feature=fig%7C6666666.294894.peg.1191) | 40831 | 42093 | + | benzoate-specific porin |
